# Interaction of NCOR/SMRT Repressor Complexes with Papillomavirus E8^E2C Proteins Inhibits Viral Replication

**DOI:** 10.1371/journal.ppat.1005556

**Published:** 2016-04-11

**Authors:** Marcel Dreer, Jasmin Fertey, Saskia van de Poel, Elke Straub, Johannes Madlung, Boris Macek, Thomas Iftner, Frank Stubenrauch

**Affiliations:** 1 University Hospital Tuebingen, Institute for Medical Virology and Epidemiology of Viral Diseases, Division of Experimental Virology, Tuebingen, Germany; 2 Proteome Center Tuebingen, University of Tuebingen, Tuebingen, Germany; University of Wisconsin Madison School of Medicine and Public Health, UNITED STATES

## Abstract

Infections with high-risk human papillomaviruses (HR-HPV) such as HPV16 and 31 can lead to ano-genital and oropharyngeal cancers and HPV types from the beta genus have been implicated in the development of non-melanoma skin cancer. HPV replicate as nuclear extrachromosomal plasmids at low copy numbers in undifferentiated cells. HPV16 and 31 mutants have indicated that these viruses express an E8^E2C protein which negatively regulates genome replication. E8^E2C shares the DNA-binding and dimerization domain (E2C) with the essential viral replication activator E2 and the E8 domain replaces the replication/transcription activation domain of E2. The HR-HPV E8 domain is required for inhibiting viral transcription and the replication of the viral origin mediated by viral E1 and E2 proteins. We show now that E8^E2C also limits replication of HPV1, a mu-PV and HPV8, a beta-PV, in normal human keratinocytes. Proteomic analyses identified all NCoR/SMRT corepressor complex components (HDAC3, GPS2, NCoR, SMRT, TBL1 and TBLR1) as co-precipitating host cell proteins for HPV16 and 31 E8^E2C proteins. Co-immunoprecipitation and co-localization experiments revealed that NCoR/SMRT components interact with HPV1, 8, 16 and 31 E8^E2C proteins in an E8-dependent manner. SiRNA knock-down experiments confirm that NCoR/SMRT components are critical for both the inhibition of transcription and HPV origin replication by E8^E2C proteins. Furthermore, a dominant-negative NCoR fragment activates transcription and replication only from HPV16 and 31 wt but not from mutant genomes encoding NCoR/SMRT-binding deficient E8^E2C proteins. In summary, our data suggest that the repressive function of E8^E2C is highly conserved among HPV and that it is mediated by an E8-dependent interaction with NCoR/SMRT complexes. Our data also indicate for the first time that NCoR/SMRT complexes not only are involved in inhibiting cellular and viral transcription but also in controlling the replication of HPV origins.

## Author Summary

Human papillomaviruses (HPV) have been shown to cause ano-genital and oropharyngeal cancers and have been also implicated in non-melanoma skin cancer. HPV have a two-stage replication cycle: in undifferentiated keratinocytes only a low level of genome replication without virus production can be observed whereas in differentiated keratinocytes high-level genome replication and virus production takes place. Previous studies have suggested that some HPV encode an E8^E2C protein that limits genome replication in undifferentiated cells. We now demonstrate that E8^E2C proteins from phylogenetically diverse HPV types interact with NCoR/SMRT corepressor complexes to limit viral transcription and genome replication. While NCoR/SMRT complexes are known to mediate the transcription repression functions of a wide variety of host transcription factors, this is the first evidence that NCoR/SMRT proteins also are involved in the repression of the replication of viral origins.

## Introduction

Human papillomaviruses (HPV) constitute one of the largest human pathogenic virus families known to date. HPV Infections can cause skin warts, ano-genital warts and papillomas derived from mucosal or cutaneous epithelium. Persistent infections with certain HPV types such as HPV16, 18, 31 and others are a necessary risk factor for the development of cervical, other ano-genital and oropharyngeal cancer [1]. In addition, infections with HPV types from the beta genus have been implicated in the development of squamous cell cancer in epidermodysplasia verruciformis patients and non-melanoma skin cancer (Beta genus papillomaviruses and skin cancer. [2].

HPV are small, naked viruses with a double-stranded, circular DNA genome of approximately 8000 bp. After infection, HPV genomes replicate as nuclear plasmids with 10 to 100 copies per cell in undifferentiated basal-like keratinocytes [3]. Viral genome copy numbers are kept constant by a random choice copy number control mechanism that is not well understood [4]. Upon differentiation of the host cell, viral genomes are amplified to several thousands of copies and as a consequence infectious virus is produced [3]. Viral proteins derived from the E1 and E2 genes function as sequence-specific DNA binding proteins and are involved in the initiation of DNA replication, control of viral transcription and segregation of viral genomes [5–7]. The E1 protein represents the viral replication initiator protein and acts as a replicative hexameric helicase [5,7]. However, despite being able to interact with the viral origin in vitro, E1 alone is not able to initiate replication in vivo and requires in addition the viral E2 protein [5–7]. E2 is a dimeric, sequence-specific DNA binding protein that recruits the E1 protein to the viral replication origin by protein-protein and protein-DNA interactions [6]. In addition, E2 can act either as a transcriptional repressor or activator which is dependent on the location of E2 binding sites (E2BS) in E2-responsive promoters and thus may also control replication by modulating viral gene expression [6]. The amino terminal domain of E2 (~200 residues) is required for the activation of replication, modulation of transcription and attachment of PV genomes to mitotic chromosomes [6]. These functions are mediated by interaction with viral E1 and cellular proteins such as Brd4 which contact E2 via highly conserved residues among PV [8–11].

In addition to E2, several human (HPV1, 5, 11, 16, 18, 31 and 33) and animal papillomaviruses (bovine papillomavirus 1, cottontail rabbit papillomavirus (CRPV)) express transcripts in which an alternative exon from the E1 gene is spliced to a 3´-exon of the E2 gene [12–21]. This gives rise to a fusion protein between the E8 gene (E9 for CRPV) which overlaps with the E1 gene and the E2C domain of E2. The corresponding proteins have been named E2C (HPV1, 11, 16, 33), E8/E2 (BPV1, HPV16), E8^E2 (HPV18), E8^E2C (HPV5, 16, 18, 31) or E9^E2C (CRPV) [12,13,15–19,21–23]. Genetic analyses of HPV5, 11, 16, 18 and 31 genomes have revealed that the loss of E8^E2C expression leads to an over replication of viral genomes in undifferentiated cells suggesting a conserved function for E8^E2C in controlling genome replication [16,19,21,22,24,25]. Transcript analyses have indicated that the E8^E2C encoding transcript is the most abundant transcript processed at the splice donor site at nt. 1301 in both undifferentiated and differentiated HPV16 wt-positive keratinocytes [20]. Studies with HPV16 have surprisingly revealed that E8- genomes not only over-replicate in undifferentiated cells but also display increased levels of viral genome copies, transcripts and late proteins in differentiated cells than the HPV16 wt [20]. This suggested that E8^E2C not only restricts viral replication and transcription in undifferentiated cells but also limits virus production.

The E2C part shared by E8^E2C and E2 proteins is responsible for DNA-binding and homo- and heterodimerization among E8^E2C and E2 proteins and thus over replication was initially thought to be due to binding-site competition between E2 and E8^E2C and/or the formation of inactive E2/E8^E2C heterodimers. However, E8^E2C proteins from HPV16, 18 and 31 act in the absence of E2 as potent transcriptional repressors from both promoter-proximal and -distal E2BS which suggested that E8^E2C proteins are active and not only competitive repressors [13,26]. Studies using HPV16 and 31 E8^E2C revealed that repression from promoter-distal E2BS is dependent upon a KWK motif in the E8 domain [20,25,26]. Interestingly, KWK or WK motifs are not only highly conserved among E8^E2C proteins of HPV11, 16, 18, 31 and 33, where E8^E2C transcripts have been identified, but can also be always found in the predicted E8 genes in a large number of HPV belonging to the alpha-papillomaviruses [13]. HPV16 or 31 genomes with mutated E8 KWK residues replicate at increased copy numbers similar to E8 knock-out genomes suggesting a critical role for the E8 domain in limiting HPV replication [24,25]. In addition to its role as a transcriptional repressor, HPV31 E8^E2C protein also inhibits the E1/E2-dependent replication of the viral origin which is also dependent upon the E8 KWK-residues and can be achieved without heterodimer formation with E2 proteins [25,27]. This strongly suggests that (1) the over replication phenotype of HPV E8^E2C mutant genomes is due to the direct interference with the E1/E2-mediated origin replication and to the repression of viral transcription and (2) that the conserved E8 domain is important for both inhibitory activities.

E8-domain dependent transcriptional repression by HPV31 E8^E2C occurs independently from other viral gene products in all human cells tested so far implying the involvement of host cell proteins [13,21,26,27]. Recent studies demonstrated that HPV31 E8^E2C does not require any of the cellular proteins recruited by E2 to mediate transcriptional repression of the viral E6/E7 promoter [28,29]. This indicated that E8^E2C proteins interact with a different set of host cell proteins to inhibit viral transcription and replication. In line with this, histone deacetylase (HDAC) inhibitors partially relieved transcriptional repression mediated by the HPV31 E8 domain and GST-pulldown experiments showed that HPV31 E8^E2C interacts in an E8-domain specific manner with cellular corepressor proteins such as HDAC 1, 2 and 3 as well as TRIM28 and SETDB1 [27]. However, a proteomic approach using HPV31 E8^E2C identified none of these proteins but rather a different set of interactors which included ARG1, BLMH, CASP14, NCoR, TBLR1 and TGM3 [28]. The functional validation demonstrated a partial release of repression by HPV31 E8^E2C by siRNAs against NCoR but not against other interactors [28]. In addition, a partial release of repression was observed with an RNAi against HDAC3 but not against other HDACs [28]. This implied that both NCoR and HDAC3 are involved in the transcriptional repression of the HPV E6/E7 promoter by HPV31 E8^E2C but that additional cellular proteins might also be involved. In line with this, HDAC inhibitors showed no effect on E8^E2C´s ability to inhibit the E1/E2-dependent replication supporting the idea of additional interaction partners [27].

NCoR and the closely related SMRT protein have been first identified as transcriptional corepressors for several unliganded and orphan nuclear receptors but have also been found to interact with other cellular transcription factors [30,31]. NCoR and SMRT proteins form salt-resistant core complexes with HDAC3, TBL1 (transducin-beta like 1), TBLR1 (TBL-related 1) and GPS2 (G protein pathway suppressor 2) [31,32]. In addition, NCoR/SMRT core complexes appear to recruit additional proteins in a transcription factor- or context-dependent manner to modulate transcription [33]. Transcription repression by NCoR/SMRT complexes has been suggested to be mainly due to HDAC3 which deacetylates histones and non-histone substrates [31,33]. However, recent data challenge this idea and indicate that deacetylase-independent mechanisms might be more important than previously thought [32,34].

NCoR has been suggested to be also a part of corepressor complexes different from NCoR/SMRT core complexes [30], leaving it open which NCoR complex is recruited by HPV E8^E2C proteins. Furthermore, the contribution of E8^E2C-interaction partners to the inhibition of the E1/E2 dependent-replication of the viral origin has not been evaluated. Since highly conserved residues of E2 mediate key interactions with viral and cellular proteins to modulate replication and transcription, it is feasible that the conserved KWK residues in the E8 domain also mediate a conserved interaction with cellular partners.

In this study we show for the first time that E8^E2C proteins also restrict the replication of HPV1 (mu-PV) and HPV8 (beta-PV) in normal human keratinocytes. The E8^E2C proteins from HPV1, 8, 16 and 31 interact with the NCoR/SMRT corepressor core complex consisting of GPS2, HDAC3, NCoR, SMRT and TBl1 and TBLR1 proteins in an E8 domain dependent manner. This interaction mediates the transcriptional repression and inhibition of E1/E2-dependent replication by E8^E2C proteins. In summary, our data reveal a novel, highly conserved role for the cellular NCoR/SMRT-corepressor complex in the control of HPV replication.

## Results

### HPV E8^E2C proteins interact with the cellular NCoR/SMRT corepressor complex

**Fig 1:**
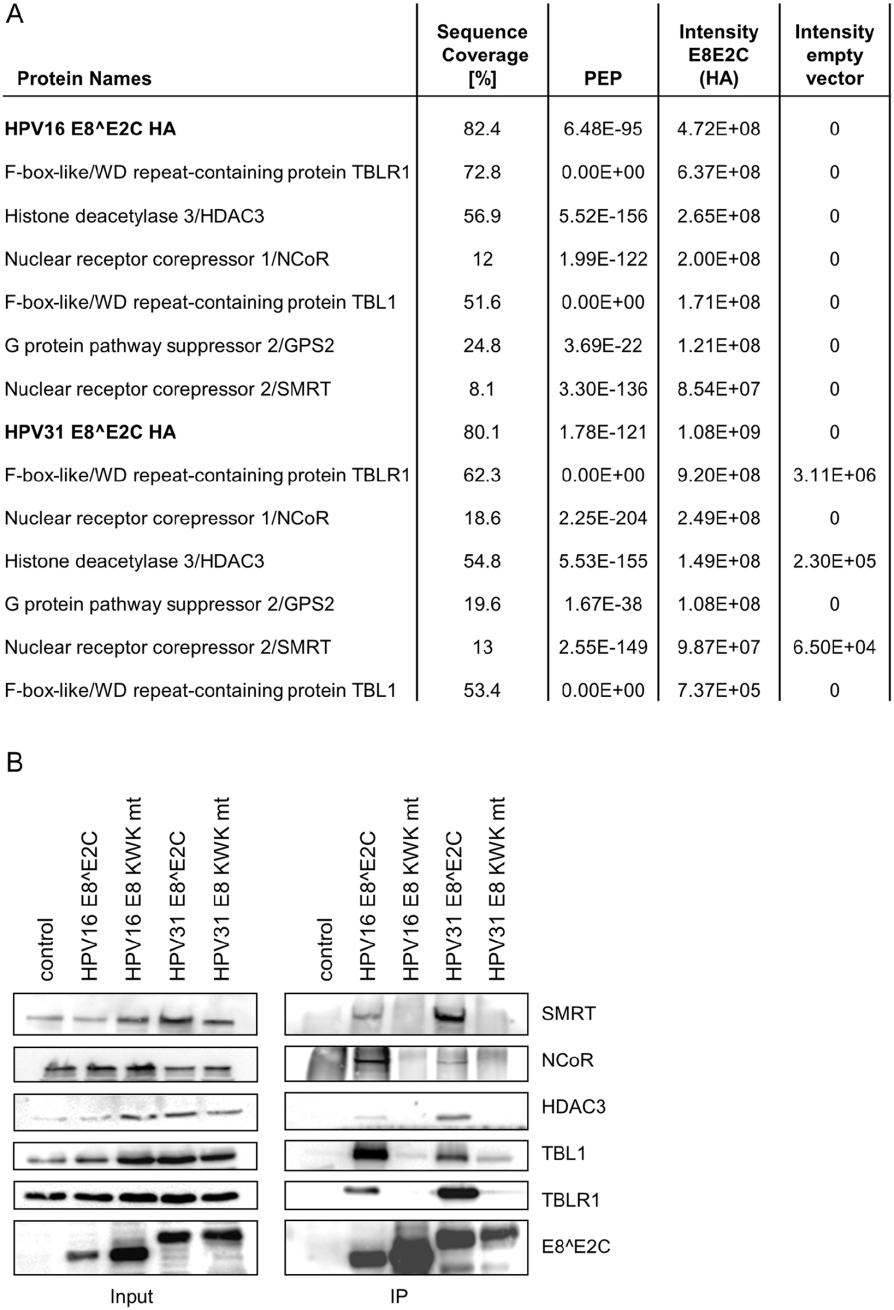
Proteomic analysis identify NCoR/SMRT complex components as E8-domain-dependent interactors of HPV16 and 31 E8^E2C proteins. (A) Common interactors of HPV16 and HPV31 E8^E2C proteins identified by NanoLC-MS/MS analysis from anti-HA immunoprecipitates from 293T/HPV16 E8^E2C-HA, 293T/HPV31 E8^E2C-HA or 293T/pIRES-puro cells (PEP = posterior error probability). (B) Co-immunoprecipitation analysis reveals an interaction of wt HPV16 and 31 E8^E2C proteins with HDAC3, NCoR, SMRT, TBL1 and TBLR1 but not with E8^E2C KWK mt proteins. Cell lysates from stable 293T cell lines were directly analyzed (input) or precipitated with α-HA antibody (IP) and subjected to immunoblot analysis with the indicated antibodies.

Previous studies have shown that the E8^E2C protein of HPV31 functionally interacts with NCoR and HDAC3 to inhibit transcription [27,28]. To get more insight into conserved HPV16 and 31 E8^E2C interactors, we established stable 293T cell lines expressing HA-tagged versions of HPV16 and 31 E8^E2C. E8^E2C proteins were immunopurified and associated proteins were identified by mass spectrometry. For both E8^E2C proteins an overlapping set of six interactors (GPS2, HDAC3, NCOR, SMRT, TBL1, TBLR1) could be identified that were either completely absent or whose intensities were greatly decreased (>100-fold) in the empty vector controls (Fig 1A). To verify these results and address if these interactors depend on a functional E8 domain, stable cell lines were established that express HA-tagged repression-deficient E8^E2C-KWK mutant (mt) proteins. Cell extracts from the empty vector control, HPV16 and 31 E8^E2C wt or HPV16 and HPV31 E8^E2C KWK mt proteins were immunoprecipitated with α-HA and subjected to immunoblot analysis. This confirmed that the wt E8^E2C proteins interact with HDAC3, NCOR, SMRT, TBL1 and TBLR1, whereas binding to the E8^E2C KWK mt proteins was not detected or greatly reduced (Fig 1B). Despite robust signals in the mass spec analysis, we were unable to confirm binding of E8^E2C proteins to GPS2 by Co-IP/immunoblot. In summary this finding confirms and extends previous studies that HPV31 E8^E2C interacts with NCoR, TBLR1 and HDAC3 [27,28]. GPS2, HDAC3, NCOR, SMRT, TBL1 and TBLR1 are known to form the stable NCoR/SMRT co-repressor complex that is recruited by unliganded nuclear receptors such as thyroid hormone receptor and retinoic acid receptor to inhibit their target genes [31,32].

**Fig 2:**
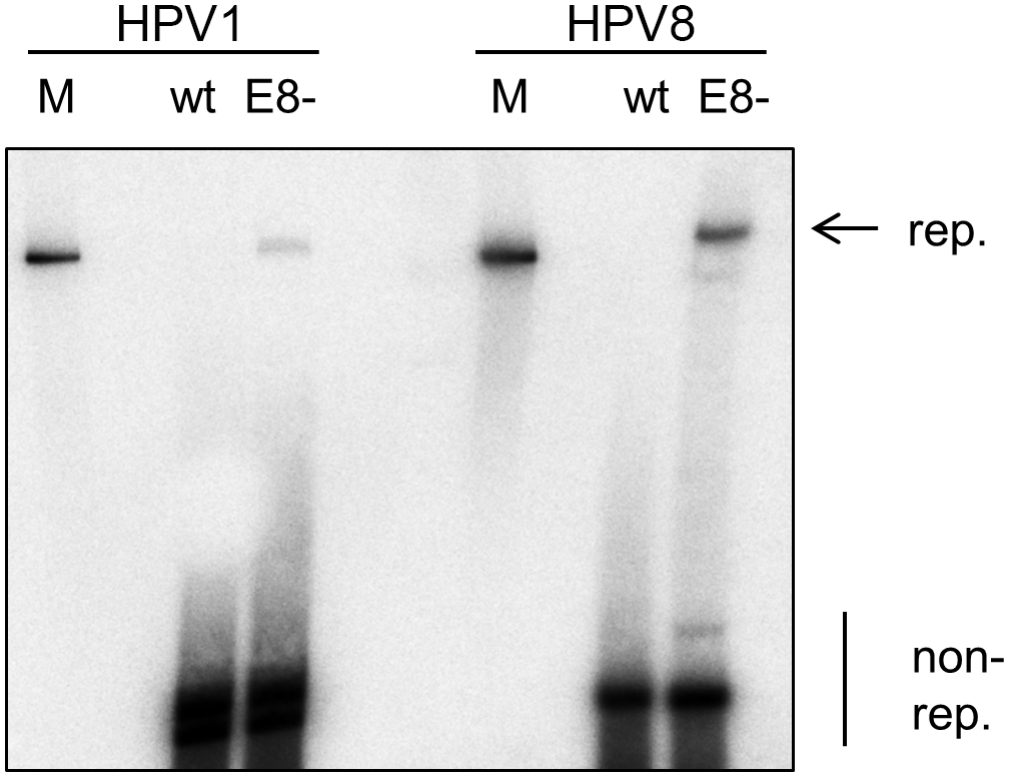
HPV1 and 8 E8- genomes replicate in normal human keratinocytes. HPV1 and 8 wt or E8- genomes were transfected into normal human keratinocytes and low-molecular weight DNA was isolated 7d p.t. and analyzed by DpnI digestion and Southern blot. Linearized HPV1 or HPV8 genomes (100pg) were used as a molecular size marker (M). The positions of DpnI-resistant, replicated (rep.) and DpnI-sensitive, non-replicated (non-rep.) genomes are indicated.

To extend our findings to E8^E2C proteins from non-alpha-PV, we first investigated the phenotype of HPV1, a mu-PV, and HPV8, a beta-PV, E8 knock-outs genomes in normal human keratinocytes (NHK). The ATG of HPV1 E8 (nt. 1200–1202) and HPV8 E8 (nt. 1312–1314) were mutated to ACG which causes a silent mutation in the overlapping E1 replication gene. Wt and mt genomes were transfected into NHK cells and low-molecular weight DNA was prepared 7d p.t, and analyzed by Southern blot after DpnI digestion to remove non-replicated genomes (Fig 2). Whereas wt HPV1 and HPV 8 genomes barely replicated, E8- genomes displayed a robust replication signal (Fig 2).

**Fig 3:**
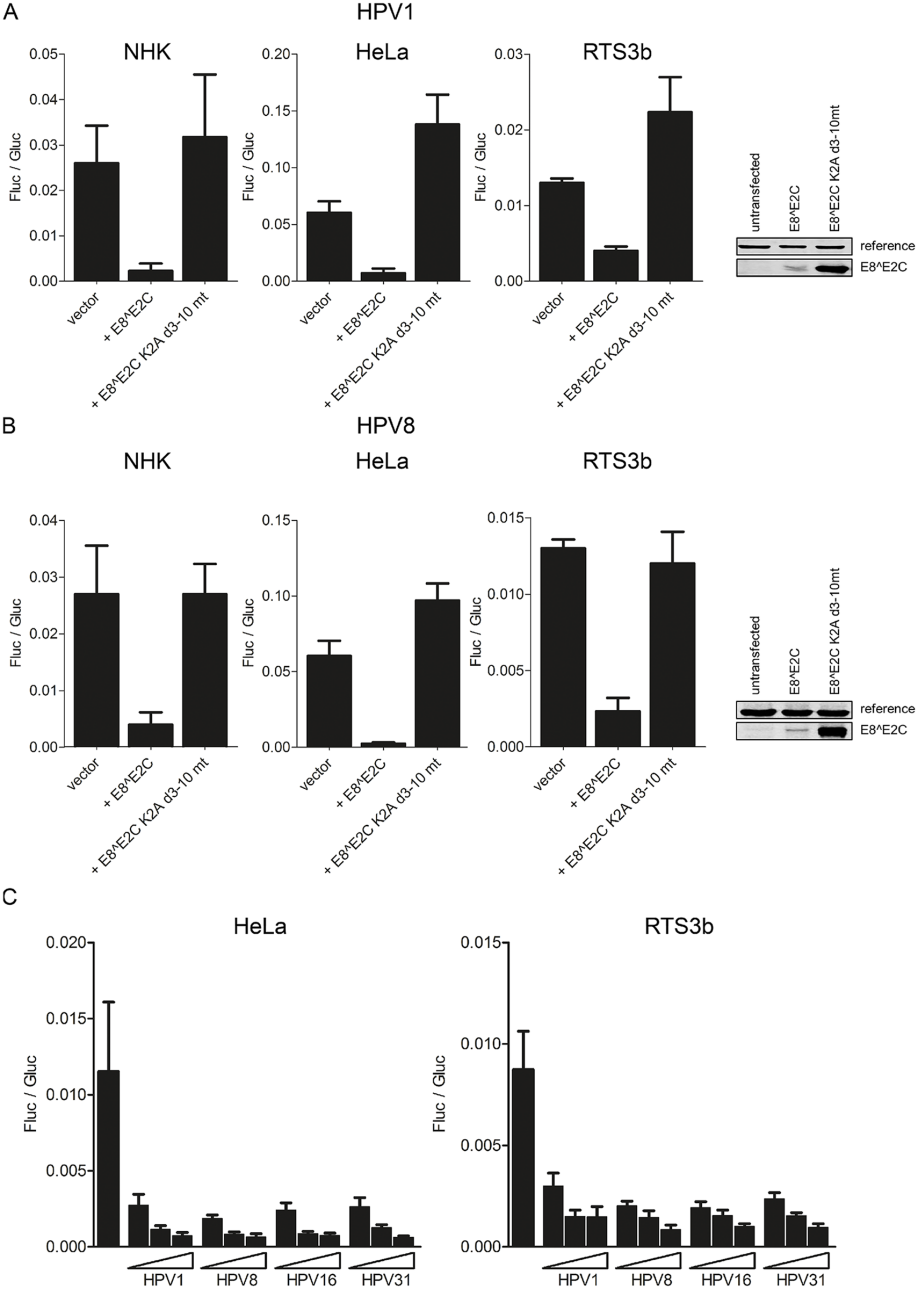
(A, B) HPV1 and E8^E2C repress transcription in an E8-domain dependent manner. NHK cells were transfected with 300ng of the pC18-Sp1-luc firefly luciferase (Fluc) reporter and 10ng of the expression vectors for HPV1 E8^E2C or E8^E2C K2A d3-10 mt or with 1ng of the expression vectors for HPV8 E8^E2C or E8^E2C K2A d3-10 mt. HeLa cells and RTS3b cells were transfected with 100ng of the reporter plasmid and with the same amounts of the expression vectors as the NHK cells. Expression vectors for HA-tagged versions of HPV1 and 8 wt or mutant E8^E2C proteins were transfected into HeLa cells and nuclear extracts were subjected to immunoblotting and analyzed with α-HA and α-KRIP1 as a reference. (C) Comparative analysis of E8^E2C repressor activities. HeLa or RTS3b cells were transfected with 100ng pC18-Sp1-luc reporter plasmid and increasing amounts of E8^E2C expression vectors (0.1, 1 or 10ng). Differences in the amount of DNA were adjusted with the empty expression vector (pSG5). pCMV-gluc (0.3ng) was used as an internal control. Values are presented as the ratio of Fluc to Gaussia luciferase (Gluc) activities. Data are derived from at least three independent experiments performed in duplicate. Error bars indicate the standard errors of the means.

To test if HPV1 and 8 E8^E2C proteins display transcriptional repression activity in an E8-domain dependent manner, expression vectors for wt E8^E2C proteins and mutant proteins without an E8 domain where lysine-2 was mutated to alanine and residues 3–10 were deleted (E8^E2C K2A d3-10 mt) were generated. Transfection experiments indicated that HPV1 and 8 E8^E2C proteins strongly repress activity from the pC18-Sp1-luc reporter plasmid which comprises four multimerized E2BS and a minimal promoter in NHK (10- and 6.8-fold), RTS3b (3- and 5.6-fold) and HeLa cells (9- and 26-fold) (Fig 3A and 3B). In contrast, repression activity of both HPV1 and 8 E8^E2C proteins was completely lost when the E8 domain was removed. Immunoblot analyses of HA-tagged E8^E2C versions revealed that the deletion of the E8 domain resulted in proteins with enhanced stability which is similar to HPV16 and 31 [24,26,28] and indicated that the loss of repression activity is not due to a decreased protein stability (Fig 3A and 3B). These data are consistent with the observations for HPV16 and 31 E8^E2C proteins that transcriptional repression is E8 domain dependent and can be observed in both normal and immortalized cells. To compare the activities of the HPV1, 8, 16 and 31 repressor proteins, titration experiments were carried out. This revealed that all E8^E2C proteins were equally able to repress transcription from the pC18-Sp1-luc reporter in both HeLa and RTS3b cell lines (Fig 3C). In summary, tthis strongly indicated that E8^E2C´s repressive function is conserved among HPV.

**Fig 4:**
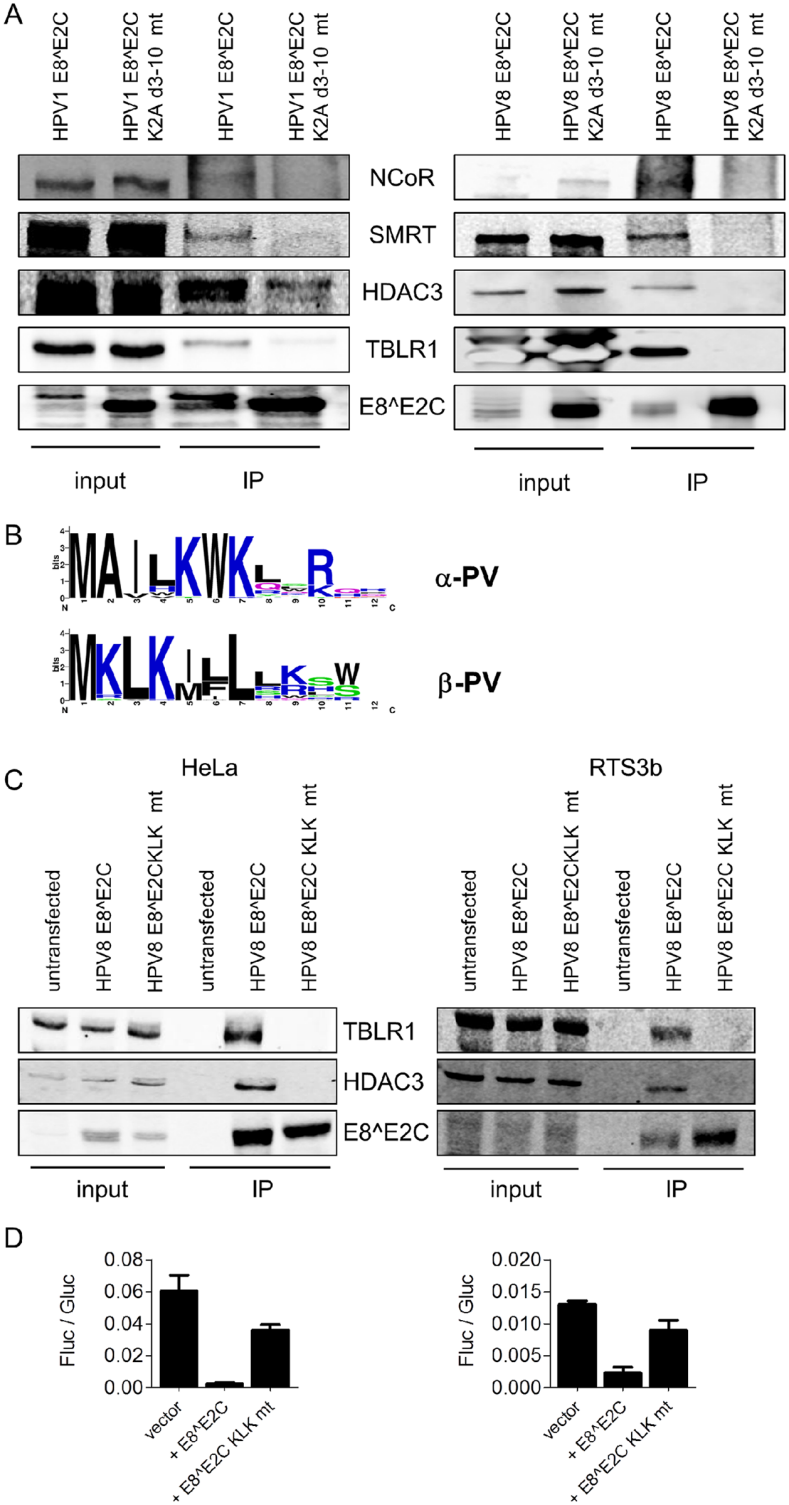
HPV1 and 8 E8^E2C proteins interact with NCoR/SMRT components in an E8-dependent manner. (A) 293T cells or HeLa cells were transfected with expression vectors for HA-tagged HPV1 and 8 E8^E2C wt or K2A d3-10 mutant proteins and whole cell lysates were directly analyzed (input) or precipitated with α-HA antibody (IP) and subjected to immunoblot analysis with the indicated antibodies. (B) E8 consensus sequences for alpha-PV (a1, 5, 6, 7, 8, 9, 10, 11 and 13 subgroups) and beta-PV (b1-6 subgroups) were obtained by extracting and aligning HPV E8^E2C sequences using the papillomavirus episteme database (http://pave.niaid.nih.gov [35]) and WebLoGo version 2.8.2 [36]. (C) The conserved KLK motif is important for the interaction of HPV8 E8^E2C with TBLR1 and HDAC3 and the transcription repression function. HeLa or RTS3b cells were transfected with expression vectors for HPV8 E8^E2C-HA wt or KLK mt—HA proteins and whole cell lysates were directly analyzed (input) or precipitated with α-HA antibody (IP) and subjected to immunoblot analysis with the indicated antibodies. (D) HeLa (left graph) or RTS3b (right graph) cells were transfected with the empty vector or HPV8 E8^E2C or E8^E2C KLK mt expression vectors (1ng), pC18-Sp1-luc (100ng) and pCMV-gluc (0.3ng). Luciferase activities were determined as described in Fig 3. Data are derived from three independent experiments and error bars indicate the SEM.

To test whether the HPV1 and HPV8 E8^E2C proteins also interact with components of the NCoR/SMRT complex, HeLa (HPV1) and 293T (HPV8) cells were transfected with wt or mutant E8^E2C expression vectors and whole cell extracts were immunoprecipitated with α-HA. Immunoblotting revealed that wt E8^E2C proteins interact with HDAC3, NCoR, SMRT and TBLR1 whereas the deletion mutants did not or in a greatly reduced manner (Fig 4A). An alignment of mu- and beta-PV E8 sequences revealed a conserved KLK motif present at residues 2–4 that resembles the functionally important KWK motif in alpha-PV (Fig 4B). To investigate this, an HPV8 E8^E2C KLK mt was generated and tested for the interaction with the NCoR/SMRT components in HeLa and RTS3b cells. The HPV8 E8^E2C KLK mt was stably expressed but failed to interact with TBLR1 and HDAC3 and only weakly inhibited transcription in HeLa and RTS3b cells (Fig 4C and 4D). The data suggest that the KLK motif is comparable to the alpha-PV KWK motif and is important for the interaction with NCoR/SMRT components. In summary, the data strongly indicate that HPV E8^E2C repressor proteins from mu- and beta-HPV also interact in an E8-dependent manner with the NCoR/SMRT complex.

### E8^E2C recruits the NCoR/SMRT complex to viral replication foci

To further validate the interaction between E8^E2C protein and NCoR/SMRT complexes in intact cells, an immunofluorescence-based assay was applied. It has been previously reported that the co-expression of HPV18 E1 and E2 proteins in HeLa cells leads to the formation of distinct foci at the origins of replication of the integrated HPV18 genomes [37]. HeLa cells were first transfected individually with epitope tagged versions of HPV31 E1, E2 and E8^E2C alone or in combination. Immunofluorescence analysis revealed that E1, E2 and E8^E2C when expressed alone have a diffuse nuclear distribution (S1 Fig). In contrast, their co-expression leads to 3–4 distinct foci that can be stained for E1/E2, E1/E8^E2C and E2/E8E2C (S1 Fig). Similar staining patterns were obtained when HPV16 E1, E2 and E8^E2C were co-expressed (S1 Fig). These data indicate that the HPV16 and 31 E1 and E2 replication proteins form nuclear foci in HeLa cells that strongly resemble HPV18 E1/E2 replication foci [37]. Furthermore these data indicate that E8^E2C repressor proteins are present in these foci.

**Fig 5:**
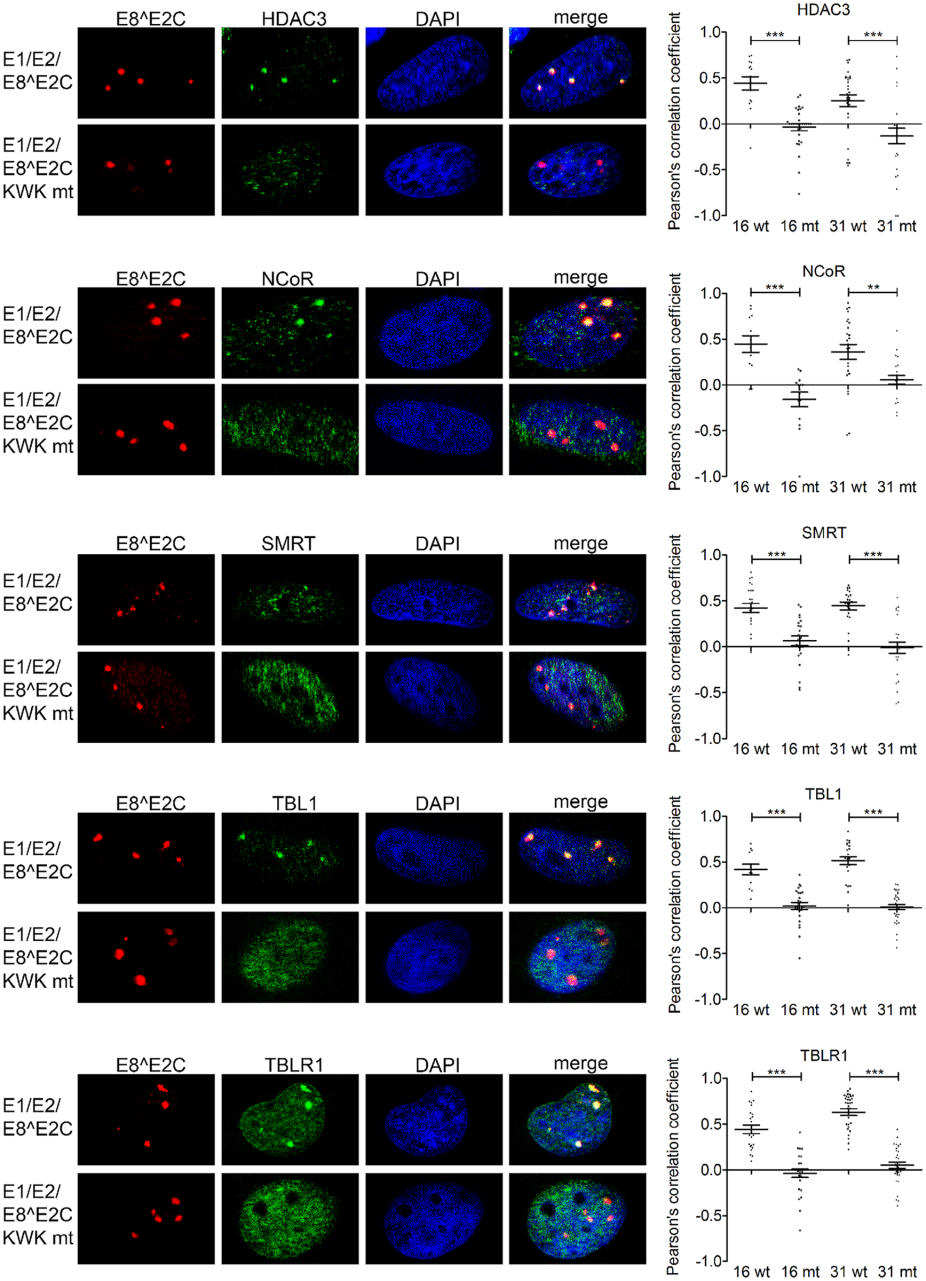
The HPV16 and 31 E8^E2C proteins recruit the NCoR/SMRT complex to viral replication foci. HeLa cells were transfected with 300ng of E1 expression vectors (pCMV neo 3xFlag-31E1 or pSG 3xFlag-16E1), 30 ng of E2 expression vectors (pSX 31 myc-E2 or pSG 16 E2) and 30 ng of the expression vectors for E8^E2C or the E8^E2C KWK mt proteins (pSG 31 E8^E2C HA/pSG 31 E8^E2C KWK mt HA or pSG 16 E8^E2C HA/pSG 16 E8^E2C KWK mt HA). Cells were stained with the indicated primary antibodies and analyzed by immunofluorescence microscopy. DNA was stained with DAPI (blue). Signals for E8^E2C (or the E8^E2C KWK mt) and NCoR/SMRT components in replication foci from 4–7 individual cells were analyzed for colocalization using optical sections and Zeiss Axiovision40 4.8.2 software. Colocalization is expressed as the Pearson´s correlation coefficient. Statistical significance between wt and mt was determined by Student´s t-test (**p<0.01; ***p<0.001).

To address if constituents of the NCoR/SMRT complex are present in such foci, HeLa cells were cotransfected with HPV31 3xflag-E1, E2-myc and E8^E2C-HA or E8^E2C-KWK mt-HA or HPV16 3xflag-E1, E2 and E8^E2C-HA or E8^E2C-KWK mt-HA. Cells were stained for E8^E2C and HDAC3, NCoR, SMRT, TBL1 or TBLR1 (Fig 5 and S2 Fig). This revealed that E8^E2C proteins localize to E1/E2-foci independent from the E8 domain. Quantification of the signals for E8^E2C and NCoR/SMRT components in replication foci revealed statistically significant colocalization of HDAC3, NCoR, SMRT, TBL1 and TBLR1 only with HPV16 or 31 E8^E2C but not with the respective E8^E2C KWK mt (Fig 5). These data indicate that NCoR/SMRT components are only recruited into replication foci in the presence of the wt E8 domain.

In addition, we tested if colocalisation occurs when an origin of replication was provided by cotransfecting HPV URR plasmids. Distinctive HPV 31 E1/E2 foci were observed whose numbers and size increased when an HPV URR plasmid was cotransfected (S3 Fig).

**Fig 6:**
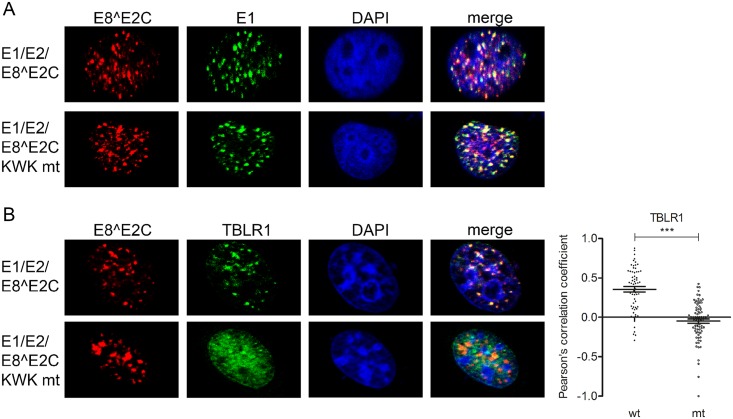
HPV 31 E8^E2C recruits NCoR/SMRT complexes to distinct nuclear foci. RTS3b were transfected with a HPV31 URR reporter plasmid and expression vectors for E1 (500 ng) (pCMV neo 3xFlag-31E1), E2 (50 ng) (pSX 31 myc-E2) and E8^E2C or the E8^E2C KWK (50 ng) (pSG 31 E8^E2C HA or pSG 31 E8^E2C KWK mt HA). Cells were stained with (A) α-flag to detect E1 and α-HA to detect E8^E2C (KWK mt) proteins or (B) α-TBLR1 and α-HA to detect E8^E2C (KWK mt) proteins. Slides were then analyzed by immunofluorescence microscopy. DNA was stained with DAPI (blue). Signals for E8^E2C (or the E8^E2C KWK mt) and TBLR1 in replication foci from 7–9 individual cells were analyzed for colocalization using optical sections and Zeiss Axiovision40 4.8.2 software. Colocalization is expressed as the Pearson´s correlation coefficient. Statistical significance between wt and mt was determined by Students t-test (***p<0.001).

The additional co-transfection of E8^E2C wt or KWK mt expression plasmids revealed that both proteins are recruited to these foci, but TBLR1 only significantly colocalizes with wt E8^E2C (Fig 6).

**Fig 7:**
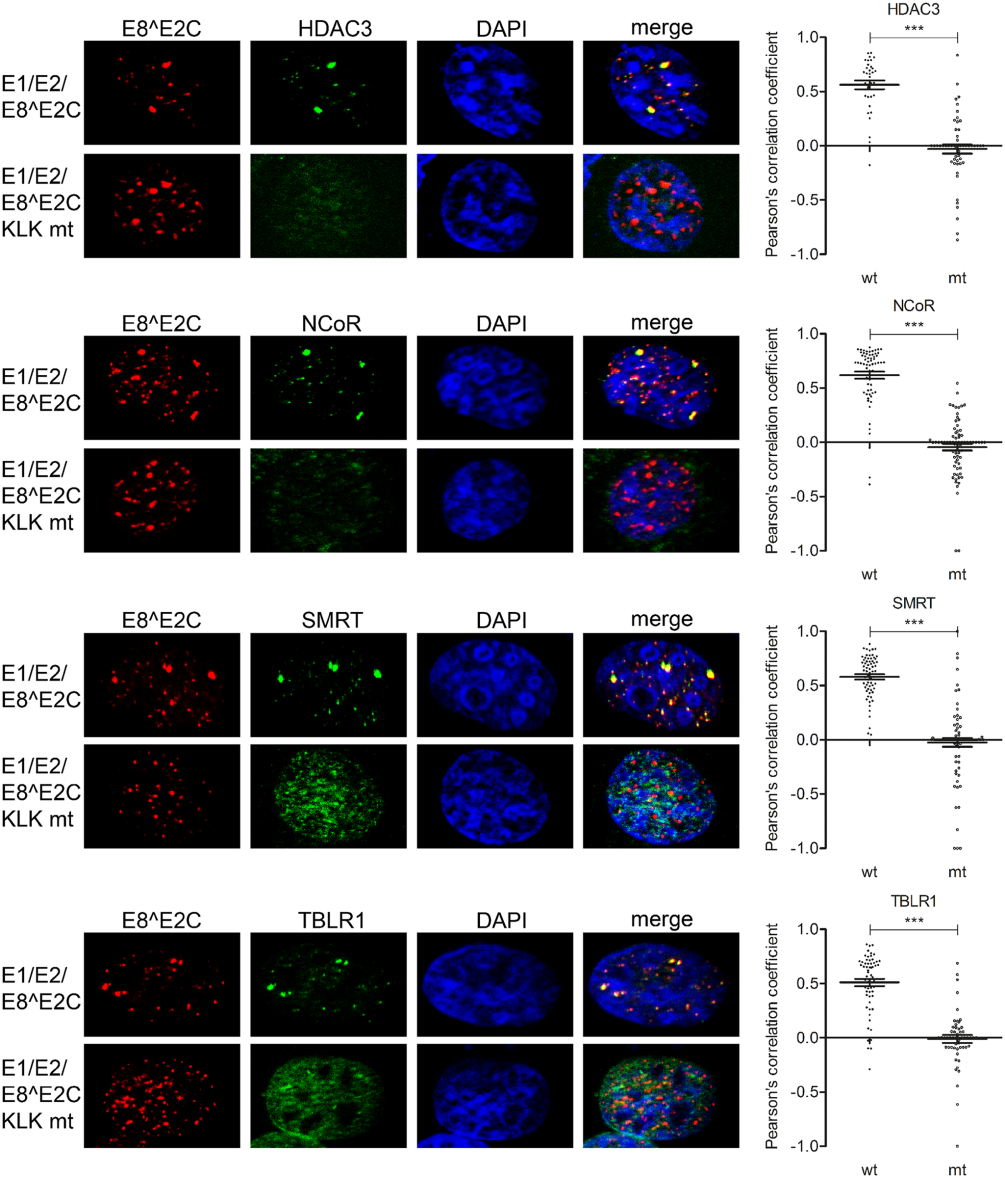
HPV 8 E8^E2C recruits NCoR/SMRT complexes to distinct nuclear foci. RTS3b were transfected with a URR reporter plasmid for HPV8 and with expression vectors for E1 (500 ng) (pCMV neo 3xFlag-8E1), E2 (50 ng) (pSG8 E2) and E8^E2C or the E8^E2C KLK mt proteins (50 ng) (pSG 8 E8^E2C HA or pSG 8 E8^E2C KLK mt HA). Cells were stained with the indicated primary antibodies and analyzed by immunofluorescence microscopy. DNA was stained with DAPI (blue). Signals for E8^E2C (or the E8^E2C KLK mt) and NCoR/SMRT complex components in foci from 6–9 individual cells were analyzed for colocalization using optical sections and Zeiss Axiovision40 4.8.2 software. Colocalization is expressed as Pearson´s correlation coefficient. Statistical significance between wt and mt was determined by Students t-test (**p<0.01; ***p<0.001).

Staining of E8^E2C upon transfection of RTS3b cells with the HPV8 URR plasmid and expression vectors for E1, E2 and E8^E2C or E8^E2C KLK mt revealed that both the HPV8 E8^E2C wt and the mt protein are present in dot-like nuclear structures (Fig 7), but recruitment of NCoR, HDAC3, SMRT and TBLR1 into these structures could only be observed in the presence of the wt E8^E2C protein (Fig 7). These results confirm that the NCoR/SMRT complex is recruited in an E8-domain dependent manner by E8^E2C proteins in vivo. Furthermore, the presence of NCoR/SMRT components in HPV16 and 31 E1/E2/E8^E2C foci suggests that the NCoR/SMRT complex is important for the repression of viral replication mediated by E8^E2C proteins.

### The NCoR/SMRT complex mediates repression of replication and transcription by HPV E8^E2C proteins

**Fig 8:**
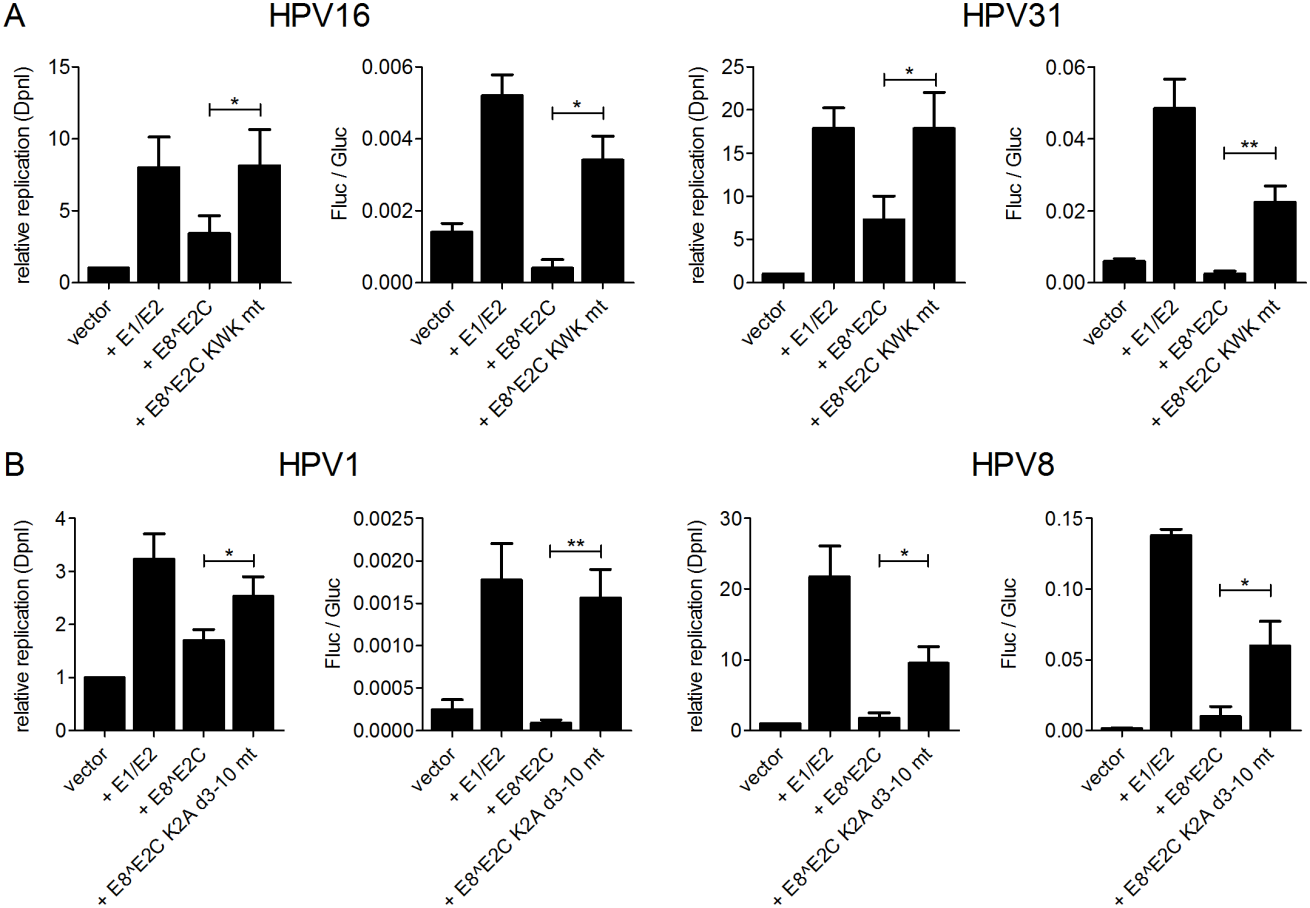
E8^E2C proteins inhibit replication in a reporter-based replication assay. (A) Replication assay for HPV16 and HPV 31. HeLa cells were transiently transfected with a reporter plasmid containing the HPV16 or 31 URR and the viral early promoter driving the firefly luciferase gene (10 ng) (pGL 16 URR luc or pGL 31 URR luc) and expression vectors for E1 (100 ng) (pSG16 E1 or pSG31 E1), E2 (10 ng) (pSG16 E2 or pSX31 E2) and E8^E2C or the E8^E2C KWK mt (HPV16: 1 ng; HPV31: 10 ng) (pSG31 E8^E2C, pSG31 E8 KWK mt, pSG16 E8^E2C, pSG16 E8 KWK mt). (B) Replication assays for HPV1 and HPV8. RTS3b cells were transfected with the HPV1 reporter plasmid and expression vectors amounts described for HPV31 (pGL 1 URR luc, pSG 1 E1, pSG 1 E2, pSG 1 E8^E2C, pSG1 E8^E2C K2A d3-10 mt). RTS3b cells were transfected with the HPV8 reporter plasmid and expression vectors as described for HPV16 (pGL 8 URR luc, pSG 8 E1, pSG 8 E2, pSG 8 E8^E2C, pSG 8 E8^E2C K2A d3-10 mt). Differences in the amount of DNA were adjusted with the empty vector (pSG5). pCMV-gluc (0.3ng) was used as an internal control. Replication activity is shown as the relative replication measured by real-time PCR after DpnI-digestion (“relative replication (DpnI)”) (reporter plasmid cotransfected with the empty expression vector = “vector” is set to 1) or as the ratio between firefly luciferase (fluc) to gaussia luciferase (gluc) activities (“Fluc/Gluc”). Data are derived from at least three independent experiments performed in duplicate. Error bars indicate the standard errors of the means. A paired two tailed t-test was used to determine statistical significance (*p<0.05, **p<0.01).

To functionally evaluate the role of the NCoR/SMRT complex during replication of the HPV origin, we made use of a luciferase based test system to measure replication activity. As previously described, the co-transfection of E1 and E2 expression vectors with an HPV16 URR reporter plasmid, which harbors the viral origin of replication and luciferase activity is driven by the major early promoter, results in an increase in luciferase activity which is mainly caused by an increase in copy number of the reporter plasmid [20]. We first validated the effects of E8^E2C in this experimental system and cotransfected HeLa cells with the respective URR plasmids and expression vectors for E1, E2 and E8^E2C or E8^E2C KWK mt from HPV16 or HPV31 and measured luciferase activity and newly replicated URR plasmids after DpnI-digestion by qPCR (Fig 8A). This revealed that the combination of HPV16 or 31 E1 and E2 induced both an increase in firefly luciferase activity (4- and 8-fold, respectively) and of newly replicated plasmids (8- and 17.9-fold, respectively). The addition of HPV16 or HPV31 E8^E2C reduced luciferase activity (13- and 20-fold, respectively) whereas the corresponding E8^E2C KWK mt did not (1.5- and 2.2-fold, respectively). Wt HPV16 or HPV31 E8^E2C inhibited the levels of newly-replicated plasmids (2.4- and 2.5-fold, respectively) whereas the E8^E2C KWK mt had no effect (Fig 8A). We then analyzed the effects on replication using HPV1 and HPV8 constructs in RTS3b cells as the HPV1 URR did not replicate efficiently in HeLa cells (Fig 8B). The co-transfection of HPV1 and HPV8 E1 and E2 expression constructs with the respective URR plasmids induced luciferase activity 7.1- and 85-fold and plasmid replication 3.2- and 21.8-fold, respectively (Fig 8B). The addition of E8^E2C repressed luciferase activity 20- and 13.7-fold and plasmid replication 1.9- and 12.8-fold, respectively. In contrast, the co-transfection with the HPV1 or HPV8 E8^E2C K2A/d3-10 mt had only minor effects on transcription (1.1- and 2.3-fold, respectively) and replication (1.3- and 2.3-fold, respectively) (Fig 8B). In summary, these data strongly support the idea that the highly conserved function of the E8 domain in HPV E8^E2C proteins is both the repression of transcription and plasmid replication.

To test the influence of NCoR/SMRT components on viral transcription and replication directly, siRNA-mediated knockdown experiments were carried out. HeLa cells were transfected with siRNA and 24h later with a mix of HPV31 reporter and expression plasmids. An efficient knock-down could only be achieved for HDAC3, NCoR, SMRT and TBLR1 but not for GPS2 or TBL1 (S4 Fig). Interestingly, the knock-down of a single complex component had no major effect on the repression activity of HPV31 E8^E2C (S4 Fig).

**Fig 9:**
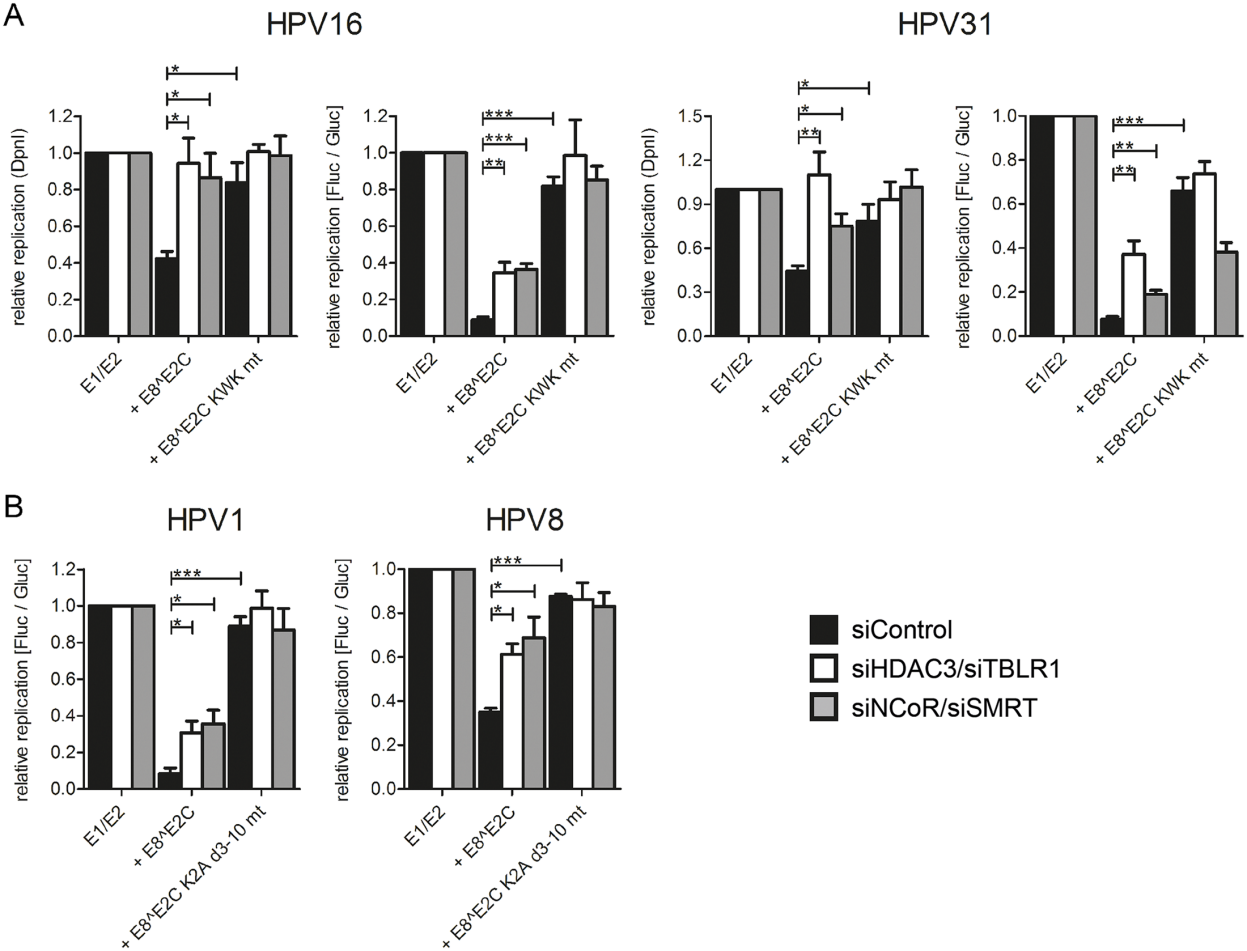
SiRNA combinations against HDAC3 and TBLR1 or NCoR and SMRT relieve the repression of replication mediated by E8^E2C proteins. (A) HeLa cells were transfected with siRNA combinations (11.7pmol of each siRNA) against HDAC3 and TBLR1 or NCoR and SMRT and 24h later reporter and expression plasmids were transfected as described in Fig 7A. (B) RTS3b cells were treated with siRNA (15pmol of each siRNA) as described for A and transfected with plasmids as described in Fig 7B. Replication activity is shown as the relative replication measured by real-time PCR after DpnI-digestion (“relative replication (DpnI)”) (“E1/E2” is set to 1) or as relative replication with “E1/E2” set to 1 derived from the ratio of Fluc to Gluc activities. Data are derived from at least three independent experiments performed in duplicate. Error bars indicate the standard errors of the means. A paired two tailed t-test was used to determine statistical significance (*p<0.05, **p<0.01, ***p<0.001).

We therefore evaluated combined knock-downs of HDAC3/TBLR1 or NCoR/SMRT in HeLa and RTS3b cells by immunoblotting which revealed that the HDAC3/TBLR1 combination also reduces the NCoR and SMRT protein levels in HeLa cells and only NCoR in RTS3b cells whereas the NCoR/SMRT combination also reduces HDAC3 amounts in both cell types (S5A Fig). Both siRNA combinations reduced the ability of HPV16 and 31 E8^E2C to repress replication to similar extents (Fig 9A). Upon knock-down of HDAC3/TBLR1 or NCoR/SMRT repression of replication was reduced to 2-fold as determined by luciferase activity and fully restored as determined by DpnI digest and qPCR (Fig 9A). Immunoblots indicated that the knock-down of HDAC3/TBLR1 had no influence on the nuclear levels of E1, E2 or E8^E2C proteins (S5B Fig). Similar to HPV16 and 31, the siRNA combinations HDAC3/TBLR1 or NCoR/SMRT reduced significantly the repression activities of HPV1 and 8 E8^E2C in RTS3b cells (Fig 9B). These data confirm that the recruitment of NCoR/SMRT complexes by E8^E2C is important for the inhibition of viral replication.

**Fig 10:**
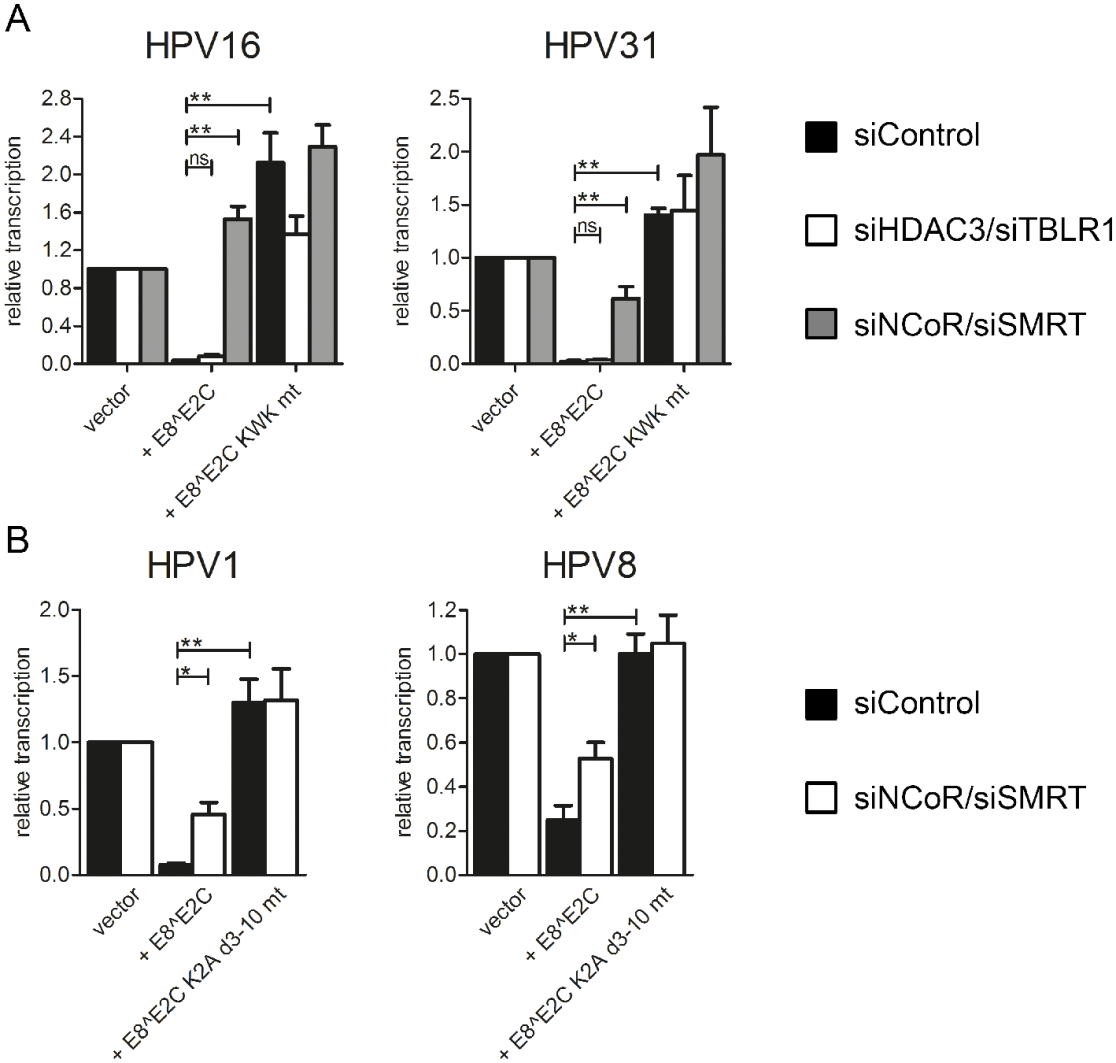
SiRNA combination against NCoR and SMRT relieves the repression of transcription mediated by E8^E2C proteins. (A) HeLa cells were transfected with siRNA combinations (11.7pmol of each siRNA) against HDAC3/TBLR1 or NCoR/SMRT and 24h later the cells were transfected with the pC18-Sp1-luc reporter (100 ng) and the expression vectors for HPV 16 or 31 E8^E2C (1 ng for HPV16, 10 ng for HPV 31) or the KWK mt proteins. (B) RTS3b cells were transfected with the siRNA combination (15pmol of each siRNA) against NCoR/SMRT and 24h later the cells were transfected with the pC18-Sp1-luc reporter (100 ng) and the expression vectors for HPV 1 or 8 E8^E2C (10 ng for HPV1, 0.2 ng for HPV 8) or the K2A d3-10 mt proteins. Differences in the amount of DNA were adjusted with the empty expression vector (pSG5). pCMV-gluc (0.3ng) was used as an internal control. Values are presented as the ratio of fluc to gluc luciferase activities. Data are derived from at least three independent experiments performed in duplicate. Error bars indicate the standard errors of the means. A paired two tailed t-test was used to determine statistical significance (*p<0.05, **p<0.01, ***p<0.001).

The activity of the pC18-Sp1-luc reporter plasmid which harbors multimerized E2BS and a minimal promoter is repressed by HPV1, 8, 16 and 31 E8^E2C in a completely E8-domain dependent manner ([24,26]; Figs 3 and 4D). In contrast to the assays using replicating HPV reporter plasmids the effects of siNCoR/siSMRT were different from siHDAC3/siTBLR1. Only siNCoR/siSMRT strongly diminished transcription repression by HPV1, 8, 16 and 31 E8^E2C, whereas siHDAC3/siTBLR1 had only a ~2-fold effect on HPV16 and 31 that was not statistically significant (Fig 10). These data indicate that NCoR and SMRT are also important for E8-dependent transcriptional repression of non-replicating plasmids. However, the differential requirements for HDAC3 and TBLR1 may indicate that these proteins are mainly required for the inhibition of replicating plasmids.

**Fig 11:**
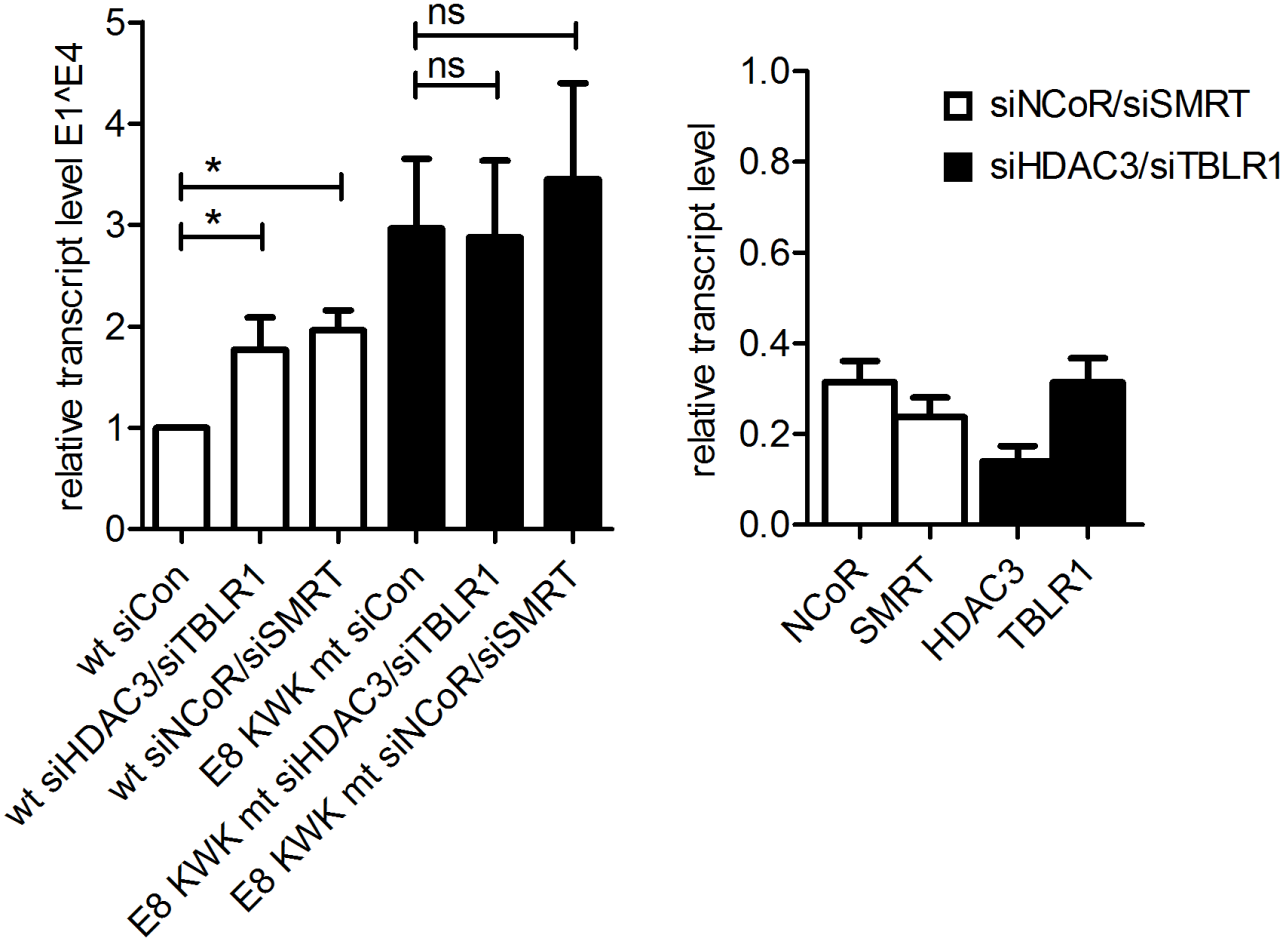
Knock-down of NCoR/SMRT components by siRNA increases transcription of HPV31 genomes. SCC13 cells were transfected with a control (siCon) or specific siRNAs (90pmol) and the next day with 1.3μg of re-ligated HPV31 wt (wt) or HPV31 E8 KWK mt (E8 KWK mt) genomes. RNA was isolated 48h later and analyzed by qPCR for the expression of spliced E1^E4, HDAC3, NCoR, SMRT, TBLR1 and PGK1 as a reference using specific primer pairs. Data are derived from four independent experiments and the values are presented relative to HPV31 wt/siControl. Error bars represent the SEM and statistical significance was tested by 1way ANOVA and Bonferroni´s multiple comparison test as a post test (*p<0.05).

To confirm our findings in the context of replicating viral genomes, we used SCC13 cells, which show high transfection efficiencies and an E8^E2C-dependent replication of HPV31 genomes [21]. Cells were first transfected with control siRNA, siHDAC3/siTBLR1 or siNCoR/siSMRT and the next day with ligated HPV31 wt or HPV31 E8 KWK mt genomes. Forty-eight h later RNA was harvested and the amounts of viral transcripts spliced at the major splice donor in E1 and the major splice acceptor in E4 (labeled E1^E4) and of HDAC3, NCoR, SMRT and TBLR1 transcripts were analyzed by qPCR (Fig 11). The average knock-down efficiencies were 69–86% NCoR/SMRT complex components (Fig 11). HPV31 E8 KWK mt genomes expressed 3-fold more E1^E4 transcripts than wt genomes in the presence of siCon. The knock-down of HDAC3/TBLR1 and NCoR/SMRT significantly induced E1^E4 transcripts 1.8-fold and 2-fold, respectively, from wt genomes whereas transcription from E8 KWK mt genomes was not significantly changed (2.9- and 3.5-fold vs. wt; Fig 11). These data confirm that NCoR/SMRT complex components repress viral transcription only in the presence of wt E8^E2C.

**Fig 12:**
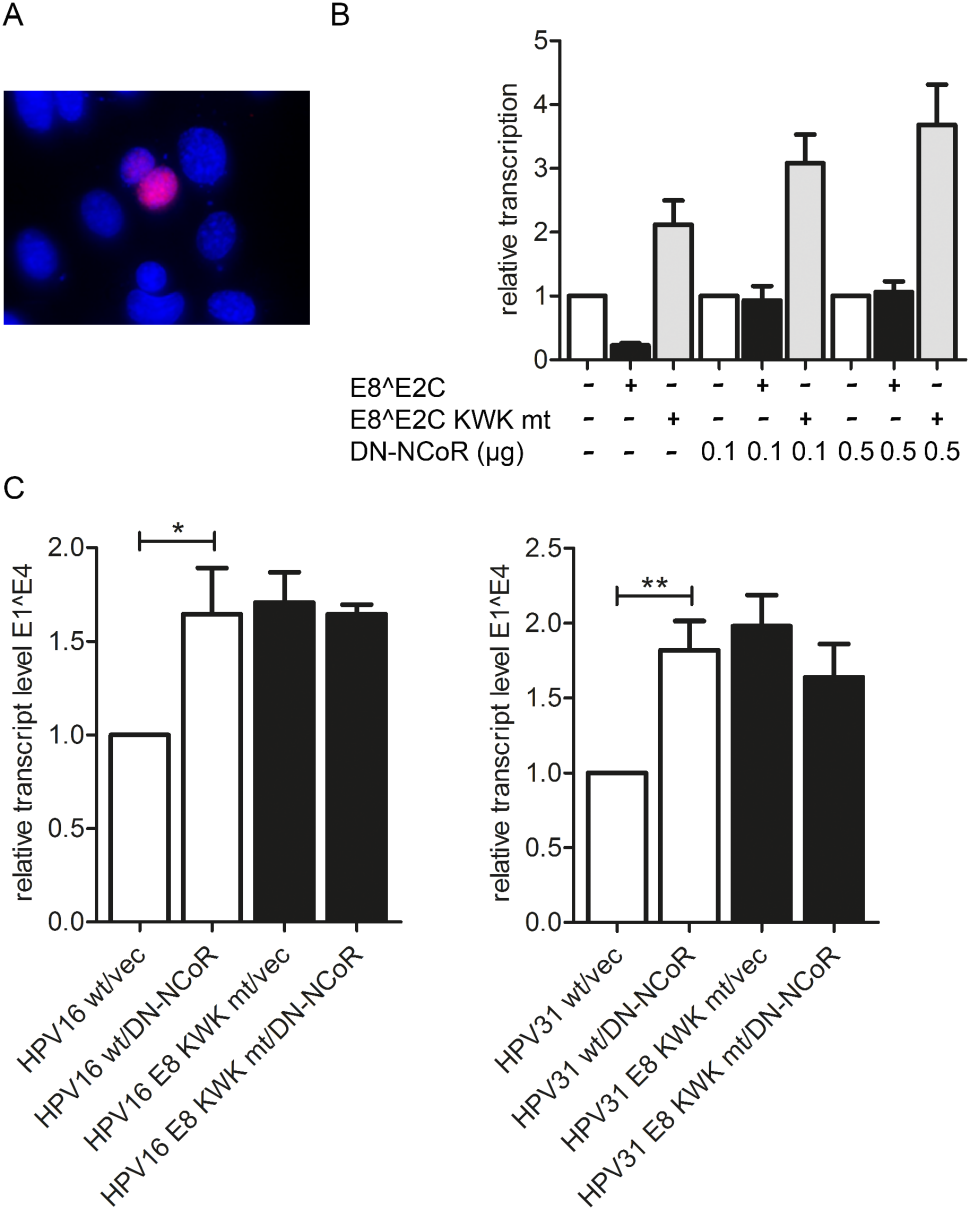
DN-NCOR activates viral transcription. (A) RTS3b cells were transfected with the pSG-DN-NCoR plasmid and stained for DN-NCoR expression using an anti-Flag antibody by indirect immunofluorescence. DAPI was used to identify cell nuclei. (B) RTS3b cells were transfected with the reporter plasmid pC18-Sp1-luc (100ng) and expression vectors for HPV31 E8^E2C, E8^E2C KWK mt (10 ng) and DN-NCoR as indicated and pCMV-Gluc as a transfection control: Data are derived from three independent transfection experiments carried out in duplicate and are presented relative to pC18-Sp1-luc/empty vector. Error bars indicate the SEM. (C) SCC13 cells were transfected with 1.3μg each of HPV16 wt, HPV16 E8 KWK mt, HPV31 wt or HPV31 E8 KWK mt genomes and 0.5μg of the empty vector (vec) or pSG DN-NCoR plasmid. RNA was analyzed by qPCR using E1^E4 and PGK1 primers. Data are derived from five independent experiments and are presented relative to HPV wt/empty vector (vec). Error bars represent the SEM and statistical significance was tested by 1way ANOVA and Bonferroni´s multiple comparison test as a post test (*p<0.05; **p<0.01).

To further extend our observations, we made use of the fact that an N-terminal fragment of NCoR has been shown to act as a dominant-negative inhibitor of Xenopus NCoR/SMRT activity [38]. This region is highly conserved among Xenopus NCoR, human NCoR and human SMRT. We therefore generated an expression plasmid for residues 1–304 of human NCoR with an N-terminal Flag tag and a nuclear localization signal (pSG DN-NCoR). We first verified nuclear expression of DN-NCoR by immunofluorescence analysis of transfected cells (Fig 12A) and then analyzed the effects of DN-NCoR on the transcriptional repression activity of HPV31 E8^E2C. The expression of DN-NCoR prevented repression of the pC18-Sp1-luc reporter by HPV31 E8^E2C in a concentration-dependent manner (5-fold increase at the highest concentration of DN-NCoR compared to the empty vector control) but had only a minor impact on the E8^E2C KWK mt (1.7-fold) (Fig 12B). To validate this in the context of HPV16 and 31 genomes, HPV16 wt, HPV16 E8 KWK mt, HPV31 wt or HPV31 E8 KWK mt genomes were cotransfected with either the empty vector or 0.5μg of the DN-NCoR expression vector into SCC13 cells. RNA was isolated 48h and analyzed by qPCR for the expression of spliced E1^E4 transcripts as described above. As expected E8 KWK mt genomes displayed 1.7- (HPV16) and 2.0-fold (HPV31) elevated transcript levels compared to the wt genomes in the presence of the empty expression vector (Fig 12C). Upon co-transfection of DN-NCoR, the transcription of wt genomes was significantly elevated 1.6- (HPV16) and 1.8-fold (HPV31), whereas transcription from the HPV16 and 31 E8 KWK mt genomes were unchanged (Fig 12C). These data strongly support the idea that NCoR/SMRT complexes are only required to inhibit viral gene expression in the presence of wt E8^E2C.

**Fig 13:**
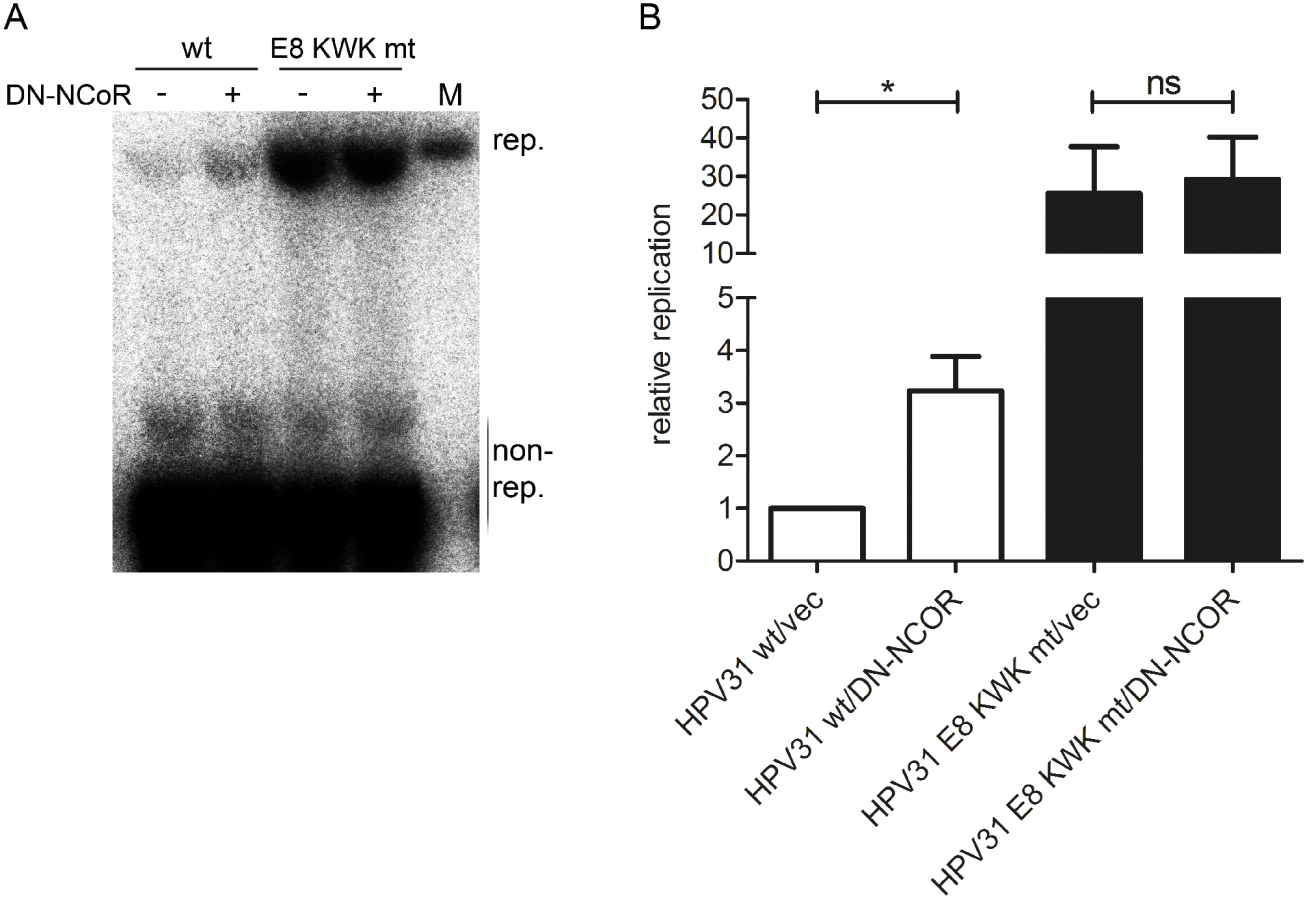
DN-NCoR activates HPV31 genome replication. SCC13 cells were co-transfected with 4μg religated HPV31 wt or E8 KWK mt genomes and 1.5μg of the empty vector pSG5 (-) or the pSG DN-NCoR plasmid. (A) Low-molecular weight DNA was isolated 72 p.t. and analyzed by Southern blotting after DpnI digestion to remove non-replicated genomes (non-rep.). M indicates 100 pg of a linearized HPV31 genome. (B) The graph shows data derived from four independent experiments which are presented relative to HPV wt/empty vector (vec). Error bars represent the SEM and statistical significance was tested by a paired Student´s t-test (*p<0.05).

We then investigated the effect of the expression of DN-NCoR on the replication of the HPV31 wt or HPV31 E8 KWK mt genomes at 72h post transfection. The cotransfection of DN-NCoR significantly increased replication of wt genomes 3.3-fold, whereas replication of the E8 KWK mt genome was unchanged (Fig 13). These data further confirm that recruitment of NCoR/SMRT complexes by E8^E2C limits HPV genome replication.

## Discussion

A small number of alpha-PV types, most notably HPV16, can cause cervical cancer and other malignancies of the ano-genital tract in persistently infected individuals. In addition, HPV that belong to the genus beta have been implicated in the development of non-melanoma skin cancer [1,2].

The replication of all PV genomes tested so far requires the viral E1 and E2 proteins, which form a complex that recognizes with high affinity the viral origin of replication in the URR. Aside from the conserved interaction with E1, E2 proteins display a highly conserved interaction with the cellular Brd4 protein [10,11,39,40]. This interaction is responsible for the ability of E2 proteins to activate transcription from synthetic promoters [39,41]. Nevertheless, the function of the E2-Brd4 interaction in the context of a viral infection is less clear and might involve the regulation of viral transcription, cellular transcription, viral genome replication and/or partitioning of viral genomes in a virus type specific manner [42].

In addition to E1 and E2, several PV have been shown to express a spliced mRNA that generates a fusion protein between the E8 ORF and the hinge and DBD/dimerization domains of E2 resulting in an E8^E2C protein. HPV16 and 31 E8^E2C knock-out genomes amplify their genomes to higher levels in normal and immortalized human keratinocytes [16,21,24], extending a concept first established for BPV1, that PV encode alternative E2 proteins that act as negative regulators of genome replication in undifferentiated cells [12,43,44]. Since the truncated BPV1 E2C protein and E8^E2C proteins harbour the DBD/dimerization domain of E2, their mechanism of action was thought to be binding site competition and formation of inactive heterodimers with E2 [45]. However, studies with HPV16 and 31 E8^E2C revealed an unexpected requirement for the E8 part of E8^E2C in limiting HPV genome replication [24,25]. Mutation of the central KWK motif, that is conserved among a large number of alpha-PV [13], resulted in E8^E2C proteins that lose transcriptional repression activity but are present at much higher amounts than wt proteins [24–26]. E8^E2C KWK mt proteins can partially diminish E1/E2-dependent replication in transfection experiments consistent with these proteins acting as binding site competitors and/or heterodimerization partners of E2 [25]. Nevertheless, HPV16 and 31 E8 KWK mt genomes over replicate to extents that are similar to E8^E2C knock-outs [24,25] which strongly suggests that the E8 part is crucial to limit HPV genome replication under physiological conditions.

Our data indicate now for the first time that E8^E2C expression is also responsible for limiting the replication of phylogenetically diverse HPV types such as HPV1 (mu-PV) and HPV8 (beta-PV) in normal human keratinocytes (Fig 2). This finding may now allow developing tissue culture replication models for beta-PV to investigate the viral life cycle and oncogenic activities of viral proteins. Interestingly, HPV1 and 8 E8^E2C proteins display properties very similar to the HPV16 and 31 proteins: they repress transcription and E1/E2-dependent replication in an E8-dependent manner (Figs 3, 4, and 8–10) emphasizing the idea that E8^E2C function is highly conserved among HPV.

Previous studies have indicated that the E8 part of HPV31 E8^E2C interacts with the host cell proteins HDAC3, NCoR and TBLR1 [27,28]. However, of these only HDAC3 and NCoR could be functionally validated by siRNA experiments [28]. By analyzing interaction partners for HPV16 and 31 E8^E2C in an unbiased manner, we were able to confirm HDAC3, NCoR and TBLR1 and found in addition GPS2, SMRT and TBL1 as common interactors. Interestingly, these proteins together form stable complexes that are known as NCoR/SMRT corepressor complexes. Consistent with these proteins being responsible for the repressive activities of E8^E2C, interactions with NCoR/SMRT complex components were greatly reduced when repression-deficient E8^E2C KWK mt proteins were analyzed in Co-IP assays. Interestingly, also HPV1 and 8 E8^E2C proteins interacted in an E8 domain-dependent manner with HDAC3, NCoR, SMRT, and TBLR1 in Co-IP experiments. The mutation of a conserved KLK motif in HPV8 E8^E2C resulted in loss of activity and of binding to HDAC3 and TBLR1. The sequence arrangement of beta-PV and HPV1 E8, which have a conserved KLK motif followed by a three residue hydrophobic stretch (MKLKhhh), resembles the HR-HPV E8, in which a stretch of three hydrophobic residues is followed by the KWK motif (MhhhKWK) (Fig 4B). The reversed order of basic and hydrophobic residues between alpha- and beta/mu-PV E8 may, however, indicate that the interactions with NCoR/SMRT complexes are not identical.

Colocalization studies confirmed that only E8^E2C wt proteins but not E8^E2C KWK or KLK mt proteins recruit HDAC3, NCoR, SMRT and TBLR1 into distinct nuclear structures that, in the case of HPV16 and 31, correspond to E1/E2-positive replication foci. The individual knock-down of HDAC3, GPS2, NCoR, SMRT or TBLR1 by siRNA showed no significant reduction of repression by HPV31 E8^E2C. In contrast, knock-down of both HDAC3 and TBLR1 or NCoR and SMRT attenuated repression of E1/E2-induced replication by all investigated E8^E2C proteins. The functional difference between individual and combinatorial knock-downs may be due to the fact that NCoR and SMRT are functional homologues and two molecules of NCoR and/or SMRT are present in corepressor complexes [32]. Therefore NCoR, NCoR/SMRT and SMRT complexes are most likely present in many cells types. Thus only the combination of siNCoR/siSMRT would target all three complexes. Furthermore, the combined knock-down of HDAC3 and TBLR1 not only reduced the targeted proteins but also decreased NCoR and SMRT levels (S5 Fig). Both siRNA combinations also specifically increased transcription from HPV31 wt genomes to similar extents whereas no activation of E8 KWK mt genomes was observed (Fig 11). Repression of the transcription reporter by HPV31 E8^E2C could be attenuated by an NCoR fragment (DN-NCoR), shown to be a dominant-negative inhibitor of NCoR/SMRT repression activity [38]. Consistent with this, co-expression of DN-NCoR activated transcription and replication from HPV16 and 31 wt genomes but not from E8 KWK mt genomes (Figs 12 and 13).

In summary our data provide strong evidence that the repressive activities of HPV E8^E2C proteins are mainly mediated by the interaction with NCoR/SMRT complexes. While NCoR/SMRT complexes have been shown to be involved in transcriptional repression of cellular genes, this is the first evidence that they also can inhibit viral replication origins.

When using a non-replicating, synthetic reporter in the absence of E1 and E2 the knock-down of NCoR/SMRT had far more pronounced effects than the HDAC3/TBLR1 knock-down on the repression by E8^E2C. As this was not the case with replicating plasmids or HPV genomes the physiological relevance of this observation remains unclear.

HDAC3 is the only known enzyme within the NCoR/SMRT core complexes and it is believed that the deacetylation of histones plays a major role in transcriptional repression by NCoR/SMRT [31]. Histone acetylation is thought to create an open chromatin environment which enables transcription activator recruitment. Furthermore, deacetylation of histones has been proposed to play a positive feed-forward role for repression by NCoR/SMRT as SMRT, NCoR, TBL1 and TBLR1 have been shown to bind preferentially to hypoacetylated histone 4 [46,47]. However, HDAC3 inhibitors only had a weak effect on transcriptional repression and showed no effect on the inhibition of E1/E2-dependent replication by HPV31 E8^E2C [27]. Furthermore, the efficient knock-down of HDAC3 alone had no discernible effect on replication repression by E8^E2C (S4 Fig). In summary, these data do not suggest that HDAC3 is critical for the repression activity of E8^E2C. Interestingly, a recent publication showed that deacetylase-dead but not NCoR/SMRT-binding deficient HDAC3 mutants could rescue hepatosteatosis and repress lipogenic genes expression in a HDAC3 knock-out mouse model [34]. These findings indicate that the deacetylase activity of HDAC3 may not always be required for repression by NCoR/SMRT complexes but rather suggest an important role for NCoR and SMRT aside from activating the deacetylase activity of HDAC3. NCoR/SMRT not only bind directly to HDAC3, GPS2, TBL1 and TBLR1 but have also been reported to interact with a large number of additional cellular proteins in a possibly context-dependent manner that contribute to the repression of transcription by NCoR/SMRT [32,48,49]. However, our proteomic analysis has not identified additional interaction partners common to HPV16 and 31 E8^E2C that are known NCoR/SMRT interactors. Future studies are required to address these questions.

Our data indicate that E8^E2C does not act by reducing the protein amounts of nuclear E1 or E2 (S5 Fig). Colocalization studies of HPV16 and 31 replication proteins revealed that the E1 protein is recruited into nuclear foci in the presence of wt E8^E2C (S1 Fig). This indicates that E8^E2C proteins also do not interfere with the localization of E1 proteins to replication foci.

In summary, our data indicate a highly conserved interaction between E8^E2C proteins and NCoR/SMRT complexes that is required to limit HPV replication.

## Materials and Methods

### Plasmid construction

The luciferase reporter plasmids pC18-SP1-luc, pGL 16URR-luc and pGL 31URR-luc have been previously described [13,21,26]. To generate pGL 1URR-luc and pGL 8URR-luc, HPV1 nt. 6939–103 or HPV8 nt. 7332–195, respectively, were amplified by PCR using the cloned HPV1 or HPV8 genomes as templates and specific primers adding XhoI (HPV1) or MluI (HPV8) restriction sites at the 5´-end and an NcoI site at the 3´-end. Amplicons were cloned into the respective restriction sites of pGL3-basic (Promega). The expression plasmids for HPV1 E1 (pSG 1 E1), HPV1 E2 (pSG 1 E2, HPV 16 E1 (pSG 16 E1, HPV16 E2 (pSG 16 E2), HPV 16 E8^E2C (pSG 16 E8^E2C), HPV 16 E8^E2C KWK mt (pSG 16 E8^E2C KWK mt), HPV 31 E1 (pSG 31 E1 and pCMV neo 3xFlag-31E1), HPV 31 E2 (pSX 31 E2), HPV 31 E8^E2C (pSG 31 E8^E2C and pSG 31 E8^E2C HA) and HPV 31 E8^E2C KWK mt (pSG 31 E8^E2C KWK mt and pSG 31 E8^E2C KWK mt HA) have been previously described [13,24,26,50–54]. To generate pIRESpuro 31E8^E2C-HA, pIRESpuro 31E8^E2C KWK mt-HA, pIRESp 16E8^E2C-HA and pIRESpuro 16E8^E2C KWK mt-HA, the respective coding sequences were PCR-amplified to add NheI and BamHI restriction sites and then cloned into pIRESpuro3 (Clontech). Plasmid pSG 3xflag-16 E1co was generated by replacing an ApaI/EcoRV fragment of pSG16 E1co [20] with an N-terminal 3xFlag epitope E1 fusion fragment (Life Technologies). To generate the expression vectors for HPV1 E8^E2C (pSG 1 E8^E2C) and HPV8 E8^E2C (pSG 8 E8^E2C) HPV1 nt. 1199-1232/3200-3797 or HPV8 nt. 1312-1343/3303-4200 were amplified using cloned HPV1 or HPV8 genomes as templates and specific primers adding EcoRI and BglII restriction sites. The resulting amplicons were cloned into EcoRI/BglII-digested pSG5 (Stratagene). Expression plasmids for E8^E2C deletion mutants (pSG 1 E8^E2C K2A d3-10 and pSG 8 E8^E2C K2A d3-10), that lack the sequence for amino acids 3 to 10 and change residue 2 from lysine to alanine, were constructed by PCR. The expression vector for the HPV8 E8^E2C KLK mt was generated by PCR using a primer that changes nucleotides 1315–1323 that code for the KLK motif to AAA. The expression plasmids for the HA-tagged HPV 1 E8^E2C proteins (pSG 1 E8^E2C HA, pSG 1 E8^E2C K2A d3-10 mt HA) were generated by overlap-extension PCR resulting in the introduction of the HA-tag between residues 30 and 31. To generate the expression plasmids for the HA-tagged HPV 8 E8^E2C proteins (pSG 8 E8^E2C HA, pSG 8 E8^E2C K2A d3-10 mt HA, PSG HPV8 E8^E2C KLK mt-HA) an oligonucleotide coding for the HA-tag was introduced into the SmaI-site of HPV8 at nt. 3539–3544. To generate the expression vectors for HPV 8 E1 (pSG 8 E1) and HPV 8 E2 (pSG 8 E2) E1 and E2 coding sequences were amplified by PCR using the cloned HPV8 genomes as template and primers adding EcoRI and BglII restriction sites. The resulting amplicons were cloned between the EcoRI/BglII sites of pSG5. Plasmid pSG 3xflag 8 E1 was generated by replacing the EcoRI/SacII fragment of pSG 8 E1 with an N-terminal 3xFlag epitope fused to a codon-optimized HPV8 E1 fragment (Life Technologies). The HPV1 wild type genome was cloned as a KpnI fragment into plasmid pBS+. To generate the respective E8- genomes the E8 ATG (HPV1 nt. 1200–1202; HPV8 nt. 1312–1314) was mutated to ACG by overlap extension PCR. This exchange is silent in the overlapping E1 gene. Plasmid pSG DN-NCoR was constructed by inserting a synthetic gene (Life Technologies) consisting of the Flag epitope, a nuclear localization signal and the coding sequence of human NCoR (residues 1–304) into the pSG5 plasmid (Stratagene). All plasmid constructs were confirmed by DNA sequencing.

### Cell culture

To generate stable 293T cell lines 3 x 10^5^ 293T cells were transfected with Fugene HD (Roche) in 60-mm dishes with 1 μg of the respective expression vectors. Cells were selected with 0.4 μg/ml puromycin for 10–12 days. Cell pools were used for subsequent experiments.

HeLa cells were maintained in DMEM (Life Technologies) and 10% fetal bovine serum (FBS). RTS3b cells were expanded in E-medium supplemented with 10% FBS [26]. Normal human keratinocytes were maintained in KSFM (Life Technologies). SCC13 cells were maintained in E-medium and 5% FBS in the presence of mitomycinC-treated NIH 3T3 J2 cells as described [21].

### Southern blot hybridization

Low molecular weight DNA from transfected NHK or SCC13 cells was isolated 7 or 3 d, respectively, post transfection. DNA was digested with DpnI and EcoRV (HPV1), BglII (HPV8) or XbaI (HPV31), and separated in 0.8% agarose gels. Blotting and hybridization to ^32^P-labeled HPV1, HPV8 or HPV31 probes were carried out as previously described [24]. After exposure of the membrane to phosphoimager screens, HPV genomes were visualized and quantitated using the AIDA software package (Raytest).

### SiRNA transfection, luciferase-based reporter assays and replication assays

For RNAi experiments cells were transfected with control siRNA (siAllstar, Qiagen) or siRNAs against NCoR (ON-TARGETplus SMARTpool, Dharmacon L-003518-00-0005), SMRT/NCoR2 (ON-TARGETplus SMARTpool, Dharmacon L-020145-01-0005), HDAC3 (ON-TARGETplus siRNA, Dharmacon J-003496-09-0010), TBLR1 (Hs_TBL1XR1_10, Qiagen SI03025925), TBL1 (ON-TARGETplus SMARTpool, Dharmacon L-012152-00-0005), GPS2 (Silencer Select, Thermo Fisher Scientific # 4392420) using HiPerfect (Qiagen) for HeLa cells and Lipofectamine RNAiMAX (Invitrogen) for RTS3b and SCC13 cells according to the manufacturer’s instructions.

For siRNA transfections followed by immunoblot analysis 3x10^5^ cells were seeded in 60mm culture dishes and lysed 72h after transfection.

For luciferase-based reporter and replication assays approximately 3x10^4^ cells were seeded in 24well dishes the day before transfection. The cells were either transfected directly with DNA with amounts as indicated in the figure legends or with siRNA followed by DNA transfection 24h later. DNA transfections were carried out using Fugene HD (Roche) and OptiMEM (Invitrogen). To analyze viral transcription, SCC13 cells were co-transfected with 1.3μg of religated HPV genomes and pSG5 or pSG DN-NCoR (0.5μg) using Fugene HD or first with the respective siRNAs and the following day with religated HPV genomes. To analyze genome replication, 4μg religated genomes and 1.5μg pSG5 or pSG DN-NCoR were used.

### Reporter assays

Luciferase-based reporter assays were carried out 48 h after DNA-transfection as previously described [26]. For reporter-based replication assays, cells were harvested 48h after DNA-transfection. The DNA was purified using a BioRobot EZ1 Workstation and the EZ1 DNA Tissue kit (Qiagen). The amount of replicated DNA was determined after DpnI digestion by quantitative real-time PCR using the primers flanking DpnI-restriction sites in the luciferase gene as described [20].

### Quantitative PCR

RNA was isolated 48h post transfection of HPV genomes using the RNeasy minikit (Qiagen). RNA (1μg) was reverse transcribed using the QuantiTect reverse transcription kit (Qiagen). Twenty-five ng of cDNA was analyzed in a LightCycler 480 using 0.3 μM gene-specific primers (PGK1 [24], HPV16 E1^E4 (16 E1E4 880/3358 F 5′-TGGCTGATCCTGCAGCAGC-3′; 16 E4 3440 R 5′-AGGCGACGGCTTTGGTATG-3′) or HPV31 E1^E4 (31 E1E4 804 F 5′-TGTTAATGGGCTCATTTGGAA-3′; 31 E4 3373 R 5′- GGTTTTGGAATTCGATGTGG-3′) and LightCycler 480 SYBR green I Master (Roche Applied Science) as previously described [24].

### Immunoblot analyses

Nuclear extracts from transfected cells, were prepared as previously described [55]. Immunoblots were performed as previously described [13]. The following primary antibodies were used at the indicated dilution: CDC47 (Thermo Fisher Scientific MS-862-P; 1:1500), c-Myc (Santa Cruz BT Sc-40 (9E11); 1:500), DYKDDDDK-Tag (Cell Signaling # 2368; 1:1000), HA-probe (Covance MMS-101P; 1:1000), HA-Tag ((C29F4), Cell Signaling # 3724; 1:1000), HA-Tag ((6E2), Cell Signaling #2367; 1:1000), HDAC3 (Cell Signaling #2632; 1:500), HSP90 (Santa Cruz sc-69703; 1:1500), myc-Tag ((71D10) Cell Signaling # 2278; 1:1000), NCoR (Bethyl Laboratories A301-145A; 1:1000), SMRT (Bethyl Laboratories 301-147A; 1:1000), TBL1 (Santa Cruz BT SC-137006; 1:500); TBLR1 (Santa Cruz BT SC-100908; 1:500). Bound antibodies were detected with anti-rabbit (polyclonal swine anti-rabbit Immunoglobulin-HRP; Dako, Hamburg, Germany, 1:3000; anti-rabbit IRDye 680RD, Li-Cor 926–68071, 1:15000; anti-rabbit IRDye 800CW, Li-CoR 926–32211, 1:15000; anti-rabbit Fluorescent TrueBlot IRDye 800, Rockland antibodies & assays # 18-3216-32, 1:10000) or anti-mouse antibodies (polyclonal rabbit anti-mouse immunoglobulin-HRP; Dako, 1:3000; anti-mouse IRDye 680RD, Li-Cor 926–68070, 1:15000; anti-mouse IRDye 800CW, Li-Cor 926–32210, 1:15000; anti-mouse Fluorescent TrueBlot DyLight 800, Rockland antibodies & assays # 18-4517-32, 1:10000) conjugated to horseradish peroxidase or to a fluorescent dye. Super-Signal West Dura reagent (Perbio Science) was used as HRP-substrate and chemiluminescent signals were recorded with a FluorSMax Imaging system (BioRad). Fluorescent signals were recorded with a Li-Cor Odyssey Fc (Li-Cor).

### Immunofluorescence microscopy

For immunofluorescence microscopy approximately 2x10^5^ HeLa or RTS3b cells were seeded and transfected on cover slips in 6well culture dishes. The cells were fixed and permeabilized with methanol/acetone for 2 minutes and incubated with primary antibodies (DYKDDDDK-Tag, Cell Signaling # 2368, 1:800; DYKDDDDK Tag (9A3), Cell Signaling # 8146, 1:1000; c-myc (9E11), Santa Cruz BT Sc-40, 1:100; HA-probe, Covance MMS-101P, 1:100; HA-Tag (C29F4), Cell Signaling # 3724, 1:1000; HA-tag (6E2), Cell Signaling #2367, 1:100; HDAC3, Cell Signaling #2632, 1:100; Myc-tag (71D10), Cell Signaling # 2278, 1:200; NCoR, Bethyl Laboratories A301-145A, 1:100; SMRT, Bethyl Laboratories 301-147A, 1:100; TBL1, Santa Cruz BT SC-137006, 1:50; TBLR1, Santa Cruz BT SC-100908, 1:50) diluted in PBS -3% BSA in a humidified chamber for 1h at room temperature. Bound antibodies were detected with anti-rabbit (anti-rabbit Alexa Fluor 555, life technologies A-21428, 1:2000; anti-rabbit Alexa Fluor 488, life technologies A-21206, 1:2000) or anti-mouse antibodies (anti-mouse Alexa Fluor 555, life technologies A-31570, 1:2000; anti-mouse Alexa Fluor 488 life, technologies A-11029, 1:2000) conjugated to a fluorescent dye. DNA was stained with DAPI. The pictures were obtained with a Zeiss Axiovert M200 microscope and the appropriate filter sets in combination with a Zeiss ApoTome to obtain optical sections with the highest E8^E2C signal while removing the out-of-focus image information. E8^E2C wt or mt positive foci of 4–9 cells were individually marked as regions of interest and the threshold for both channels was determined by a statistical method described by Costes et al. [56] implemented in the Axiovision40 4.8.2 software module. Co-localization of E8^E2C with the components of the NCoR complex is shown as the Pearson’s correlation coefficient.

### Co-Immunoprecipitation experiments

Three x 10^8^ cells (NanoLC-MS/MS analysis) or 1.5 x 10^7^ cells (immunoblot analysis) were harvested and then lysed in IP-buffer (50mM HEPES pH7.5, 150mM NaCl, 10% (v/v) glycerol, 5mM EDTA, 1mM DTT, protease and phosphatase inhibitors or 50mM HEPES pH7.9, 150mM NaCl, 0.3% (v/v) Igepal 630, 1mM DTT, protease and phosphatase inhibitors). Immunoprecipitation was carried out using magnetic anti-HA-beads (Miltenyi Biotech). Beads were washed with IP-buffer using μMACS columns and μMACS Separator (Miltenyi Biotech). Bound proteins were eluted in 4 x SDS gel loading buffer (Carl Roth) heated to 95°C and an aliquot analyzed by HA-immunoblotting to monitor the efficiency of the IP.

### NanoLC-MS/MS analysis

Complete protein eluates of the pull-down experiments were submitted to a short run (1cm in length) on 1D SDS PAGE (NuPAGE 12% precast Bis/Tris gels, Invitrogen) for purification. The proteins were visualized by staining using the Novex Colloidal Blue Staining Kit (Invitrogen) according to the manufacturer’s instructions and the corresponding gel sectors were excised and subjected to tryptic in-gel digestion as described previously [57]. The resulting peptide mixtures were desalted with C_18_ Stage Tips [58] before LC/MS measurement. All LC-MS analysis were performed on a nanoLC (Easy-nLC, Thermo Fisher Scientific, formerly Proxeon Biosystems) coupled to a LTQ-Orbitrap-XL (Thermo Fisher Scientific). Chromatographic separation of the peptides was performed on a 15-cm fused silica emitter of 75-mm inner diameter (New Objective), in-house packed with reversed-phase ReproSil-Pur C18-AQ 3-mm resin (Dr. Maisch GmbH). The peptide mixtures were injected onto the column in HPLC solvent A (0.5% acetic acid) at a flow rate of 500 nL/min and subsequently eluted with a 124-min segmented gradient of 5%–80% HPLC solvent B (80% ACN in 0.5% acetic acid) at a flow rate of 200 nL/min. MS data acquisition was conducted in the positive ion mode. The mass spectrometer was operated in the data-dependent mode to automatically switch between MS and MS/MS acquisition. Survey full-scan MS spectra were acquired in the mass range of m/z 300–2000 in the orbitrap mass analyzer at a resolution of 60,000. An accumulation target value of 10^6^ charges was set and the lock mass option was used for internal calibration. The 10 most intense ions were sequentially isolated and fragmented in the linear ion trap using collision-induced dissociation (CID) at the ion accumulation target value of 5000 and default CID settings. The ions already selected for MS/MS were dynamically excluded for 90 sec. The resulting peptide fragment ions were recorded in the linear ion trap.

### Data processing and analysis

MS data were processed using the MaxQuant software suite (1.0.14.3) [59]. The Mascot search engine v.2.2 (Matrix Science) was utilized to search the generated peak lists against a target-decoy database [60] consisting of the IPI human database (ipi.HUMAN.v3.73) containing 89652 protein entries plus the sequences of the viral proteins used in the experiment and 262 commonly observed contaminants. In the database search, carbamidomethylation (Cys) was set as fixed modification, whereas oxidation (Met) and acetylation (protein N termini) were set as variable modifications. The mass tolerances for precursor and fragment ions were set to 7 parts per million (ppm) and 0.5 Dalton, respectively. Identified MS/MS spectra were further processed by MaxQuant for statistical validation and quantitation of peptides, sites, and protein groups [61]. False discovery rates [60] were set to 1% at site, peptide, and protein group level.

### Accession numbers

HPV1 E1 (VE1_HPV1A; P03111), HPV1 E2 (VE2_HPV1A; P03118), HPV8 E1 (VE1_HPV08; P06420), HPV8 E2 (VE2_HPV08; P06422), HPV16 E1 (VE1_HPV16; P03114), HPV16 E2 (VE2_HPV16; P03120), HPV31 E1 (VE1_HPV31; P17382), HPV31 E2 (VE2_HPV31; P17383), GPS2 (GPS2_HUMAN; Q13227), HDAC3 (HDAC3_HUMAN; O15379), NCoR (NCOR1_HUMAN; O75376), SMRT (NCOR2_HUMAN; Q9Y618), TBL1 (TBL1X_HUMAN; O60907), TBLR1 (TBL1R_HUMAN; Q9BZK7).

## Supporting Information

S1 Fig HPV16 and 31 E1, E2 and E8^E2C proteins colocalize in distinct nuclear foci. (A) HeLa cells were transfected with 300ng of the HPV31 E1 expression vector (pCMV neo 3xFlag-31E1), 30ng of the HPV31 E2 expression vector (pSX 31 myc-E2) or 30ng of the HPV31 E8^E2C expression vector (pSG 31 E8^E2C HA). Cells stained with the indicated primary antibodies and analyzed by Immunofluorescence microscopy. DNA was stained with DAPI (blue). HeLa cells were transfected with combinations of HPV31 (B) or HPV16 (C) E1, E2 or E8^E2C expression vectors and stained as described above.
(TIF)

S2 Fig The HPV16 E8^E2C protein recruits the NCoR/SMRT complex to viral replication foci. HeLa cells were transfected with 300ng of E1 expression vector (pSG 3xFlag-16E1), 30 ng of E2 expression vector (pSG 16 E2) and 30 ng of the expression vector for E8^E2C or the E8^E2C KWK mt protein (pSG 16 E8^E2C HA or pSG 16 E8^E2C KWK mt HA). Cells were stained with the indicated primary antibodies and analyzed by immunofluorescence microscopy. DNA was stained with DAPI (blue).
(TIF)

S3 Fig HPV31 E1 and E2 colocalize in distinct nuclear foci in HPV-negative cells when a viral replication origin is cotransfected. RTS3b were transfected with the ori-containing reporter plasmid pGL 31URR luc (500ng) and expression vectors for HPV 31 E1 (500 ng) (pCMV neo 3xFlag-31E1) and E2 (50 ng) (pSX 31 myc-E2). Cells were stained with the indicated primary antibodies and analyzed by immunofluorescence microscopy. DNA was stained with DAPI (blue).
(TIF)

S4 Fig RNAi against single NCoR/SMRT complex components has no major effect on the repression activity of HPV31 E8^E2C. HeLa cells were transfected with siRNAs (11.7pmol) against NCoR, SMRT, HDAC3, TBL1, TBLR1 and GPS2. Twenty-four h later reporter and expression plasmids were transfected as described in Fig 7. Replication activity is shown as the relative replication measured by firefly luciferase (fluc) activity. Replication activated by E1/E2 is set to 1. Data are derived from at least three independent experiments performed in duplicate. Error bars indicate the SEM. To control knockdown efficiency nuclear extracts of siRNA transfected cells were subjected to immunoblotting and analyzed with the indicated antibodies.
(TIF)

S5 Fig Effects of siRNAs against NCoR/SMRT complex components. (A) HeLa or RTS3b were transfected with siRNA combinations (80pmol of each siRNA in HeLa cells and 166pmol of each siRNA in RTS3b cells) against HDAC3 and TBLR1 or against NCoR and SMRT. (B) HeLa cells were either not transfected (untransfected) or transfected with the siControl or the combination against HDAC3 and TBLR1. Twenty-four h later the cells were transfected with expression vectors for HPV 31 Flag-E1 (500 ng), myc-E2 (500 ng) and HA-E8^E2C or the KWK mt. Nuclear extracts were prepared and analyzed by immunoblotting with the indicated antibodies. KRIP1 was used as the nuclear reference to perform a quantification of Immunoblot-signals with the protein levels of the “siControl” sample (for A) or the “w/o RNAi” sample (for B) set to 100%.
(TIF)
