# Supplementary figures and images for: Interaction of NCOR/SMRT Repressor Complexes with Papillomavirus E8^E2C Proteins Inhibits Viral Replication
Source: PLoS Pathog. 2016 Apr 11;12(4):e1005556. doi: 10.1371/journal.ppat.1005556 (PMC4827801; doi:10.1371/journal.ppat.1005556)

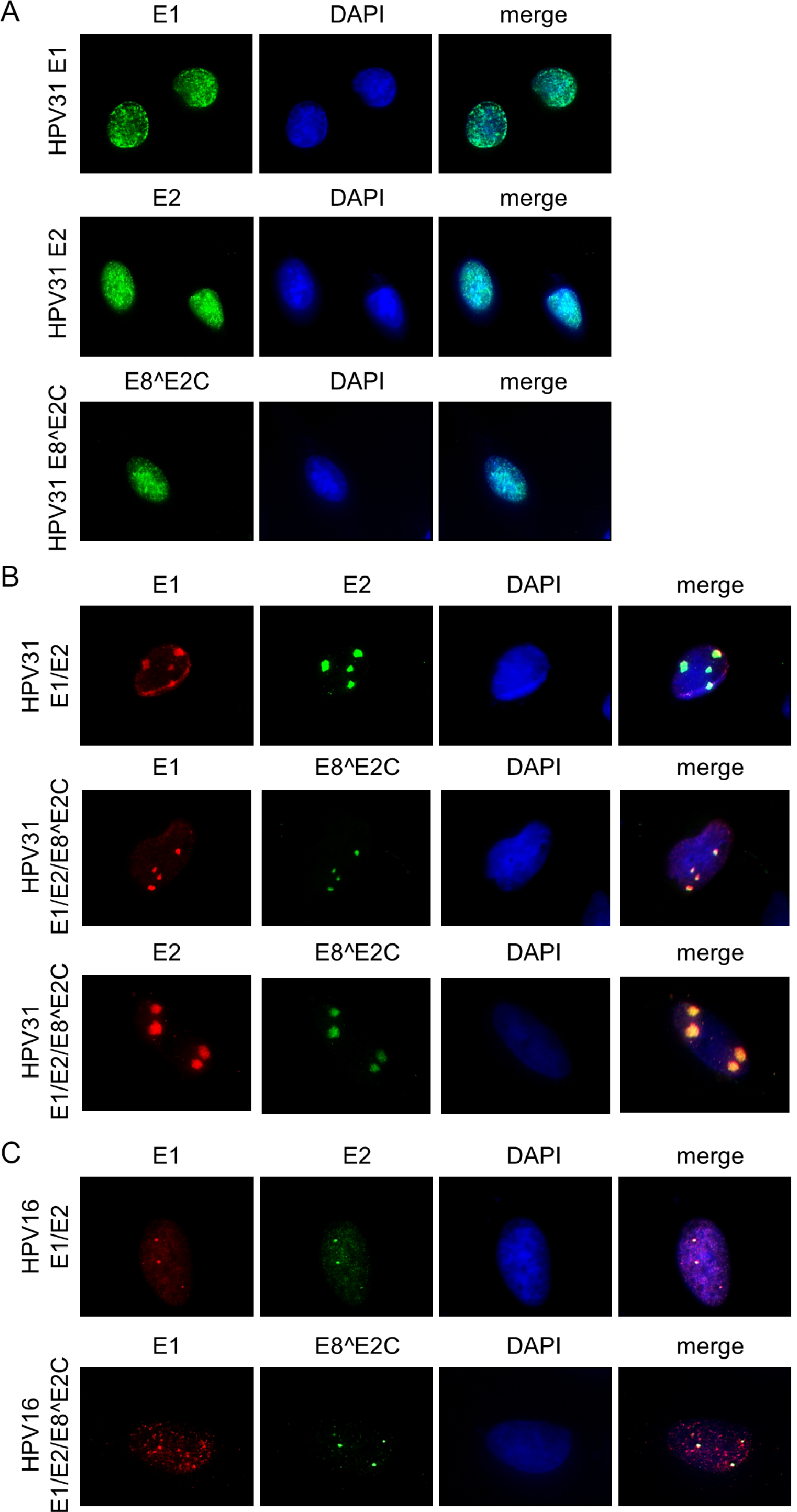

Supplement: S1 Fig — (A) HeLa cells were transfected with 300ng of the HPV31 E1 expression vector (pCMV neo 3xFlag-31E1), 30ng of the HPV31 E2 expression vector (pSX 31 myc-E2) or 30ng of the HPV31 E8^E2C expression vector (pSG 31 E8^E2C HA). Cells stained with the indicated primary antibodies and analyzed by Immunofluorescence microscopy. DNA was stained with DAPI (blue). HeLa cells were transfected with combinations of HPV31 (B) or HPV16 (C) E1, E2 or E8^E2C expression vectors and stained as described above. (TIF) [file ppat.1005556.s001.tif]

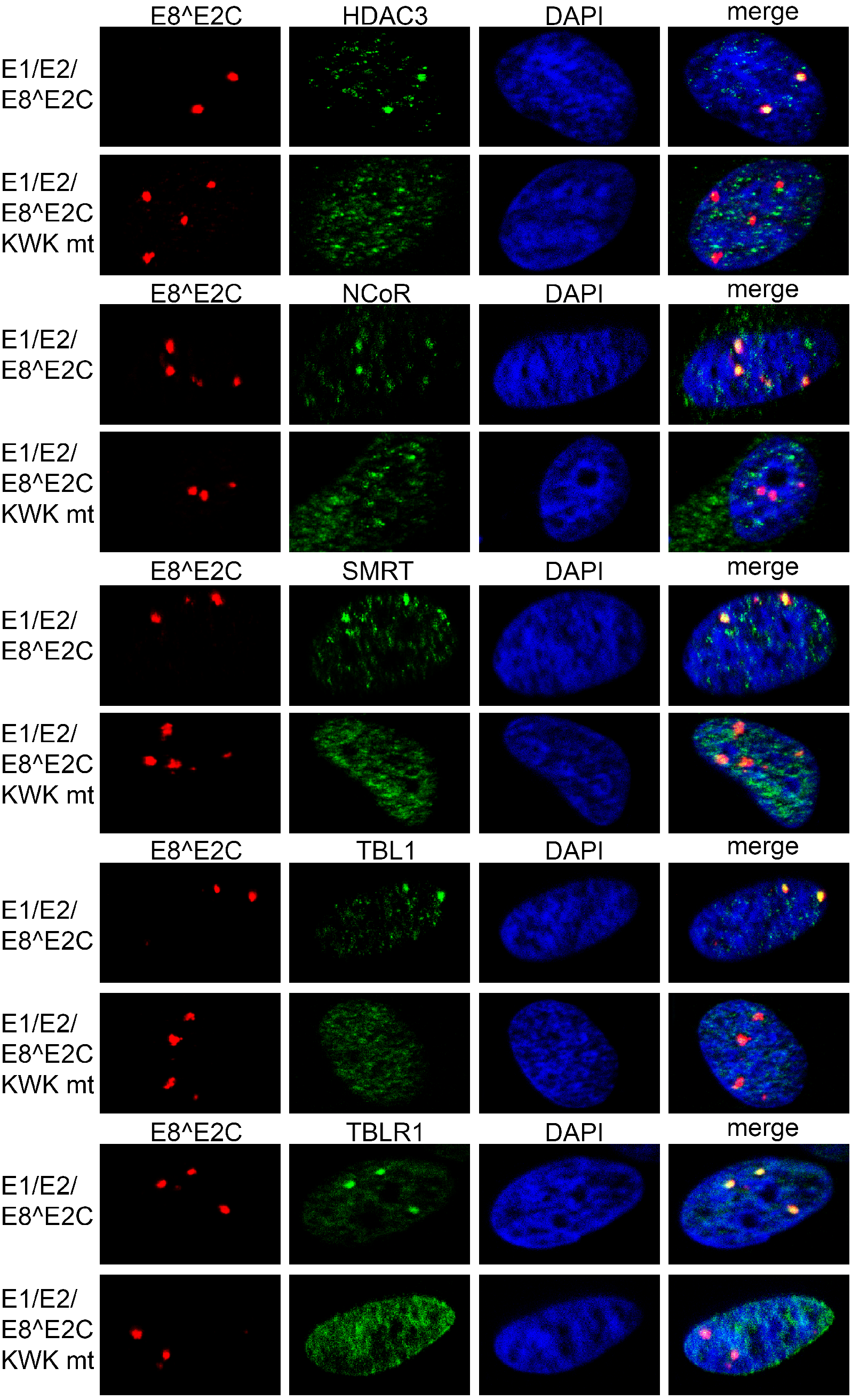

Supplement: S2 Fig — HeLa cells were transfected with 300ng of E1 expression vector (pSG 3xFlag-16E1), 30 ng of E2 expression vector (pSG 16 E2) and 30 ng of the expression vector for E8^E2C or the E8^E2C KWK mt protein (pSG 16 E8^E2C HA or pSG 16 E8^E2C KWK mt HA). Cells were stained with the indicated primary antibodies and analyzed by immunofluorescence microscopy. DNA was stained with DAPI (blue). (TIF) [file ppat.1005556.s002.tif]

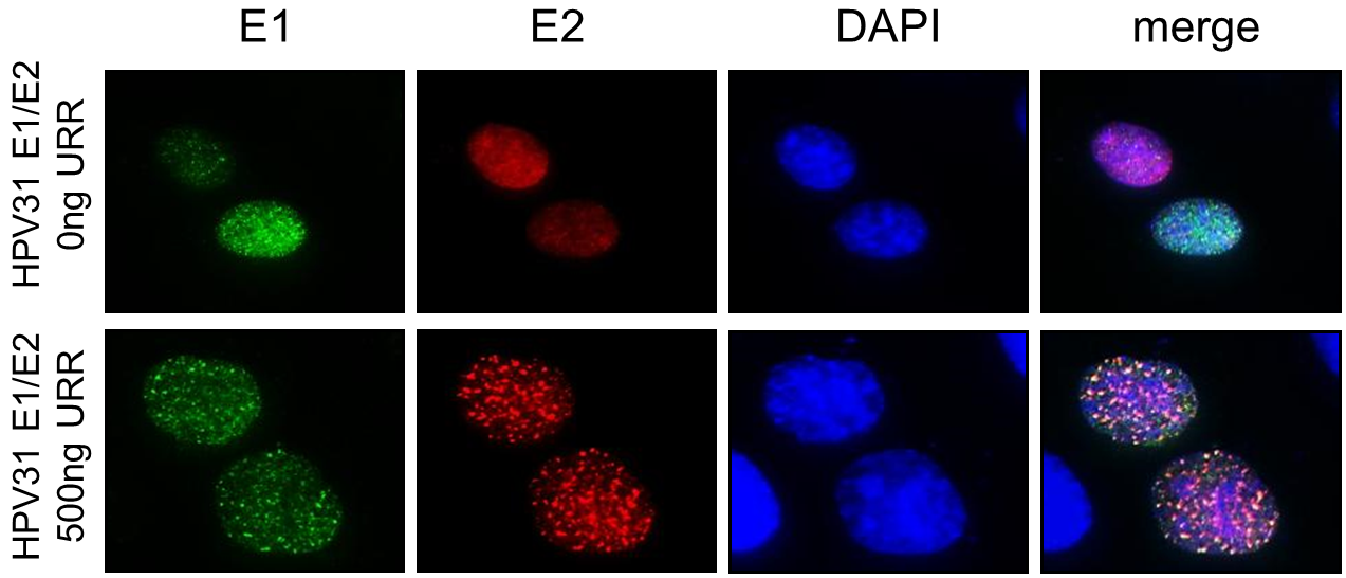

Supplement: S3 Fig — RTS3b were transfected with the ori-containing reporter plasmid pGL 31URR luc (500ng) and expression vectors for HPV 31 E1 (500 ng) (pCMV neo 3xFlag-31E1) and E2 (50 ng) (pSX 31 myc-E2). Cells were stained with the indicated primary antibodies and analyzed by immunofluorescence microscopy. DNA was stained with DAPI (blue). (TIF) [file ppat.1005556.s003.tif]

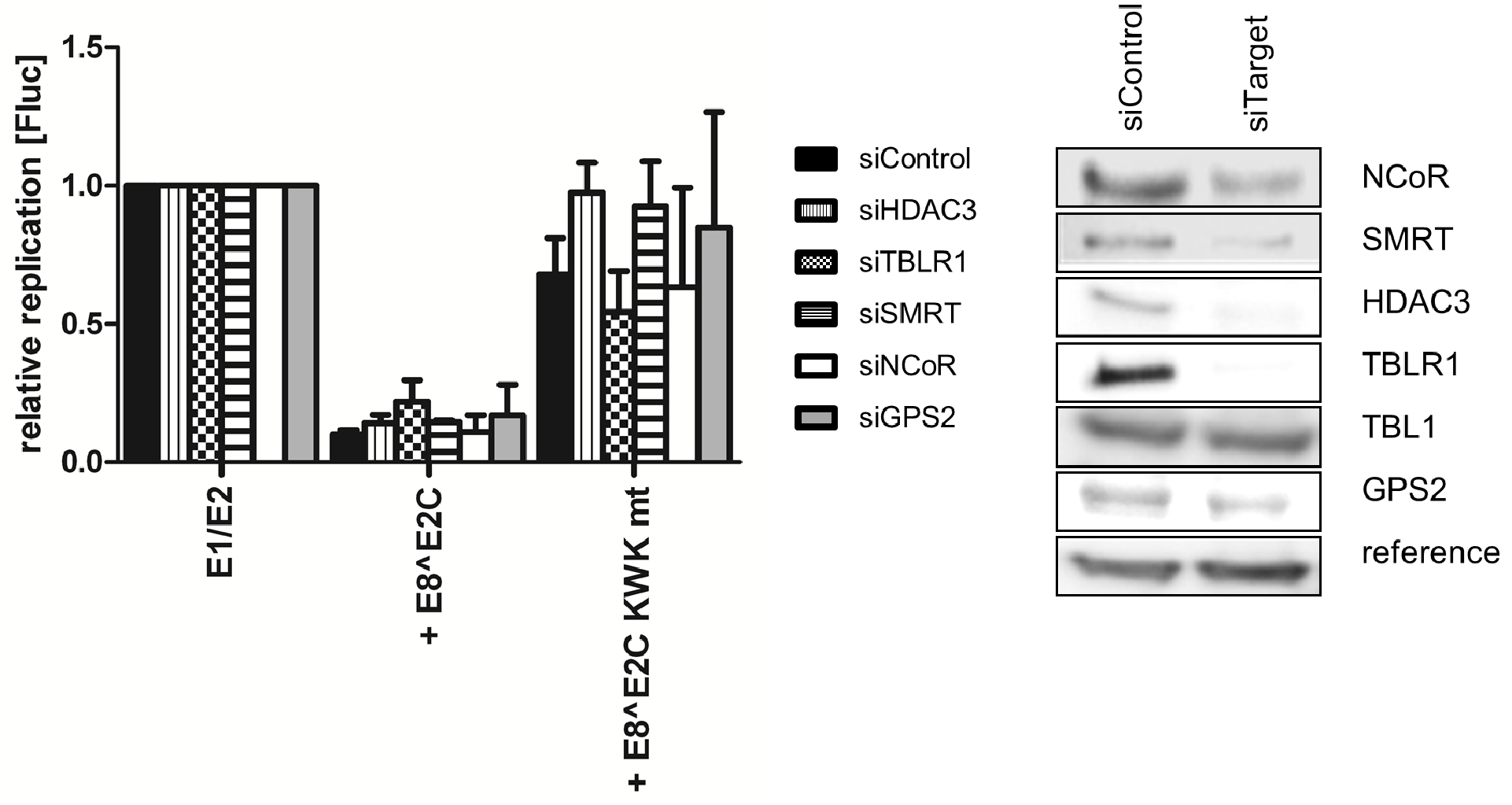

Supplement: S4 Fig — HeLa cells were transfected with siRNAs (11.7pmol) against NCoR, SMRT, HDAC3, TBL1, TBLR1 and GPS2. Twenty-four h later reporter and expression plasmids were transfected as described in Fig 7. Replication activity is shown as the relative replication measured by firefly luciferase (fluc) activity. Replication activated by E1/E2 is set to 1. Data are derived from at least three independent experiments performed in duplicate. Error bars indicate the SEM. To control knockdown efficiency nuclear extracts of siRNA transfected cells were subjected to immunoblotting and analyzed with the indicated antibodies. (TIF) [file ppat.1005556.s004.tif]

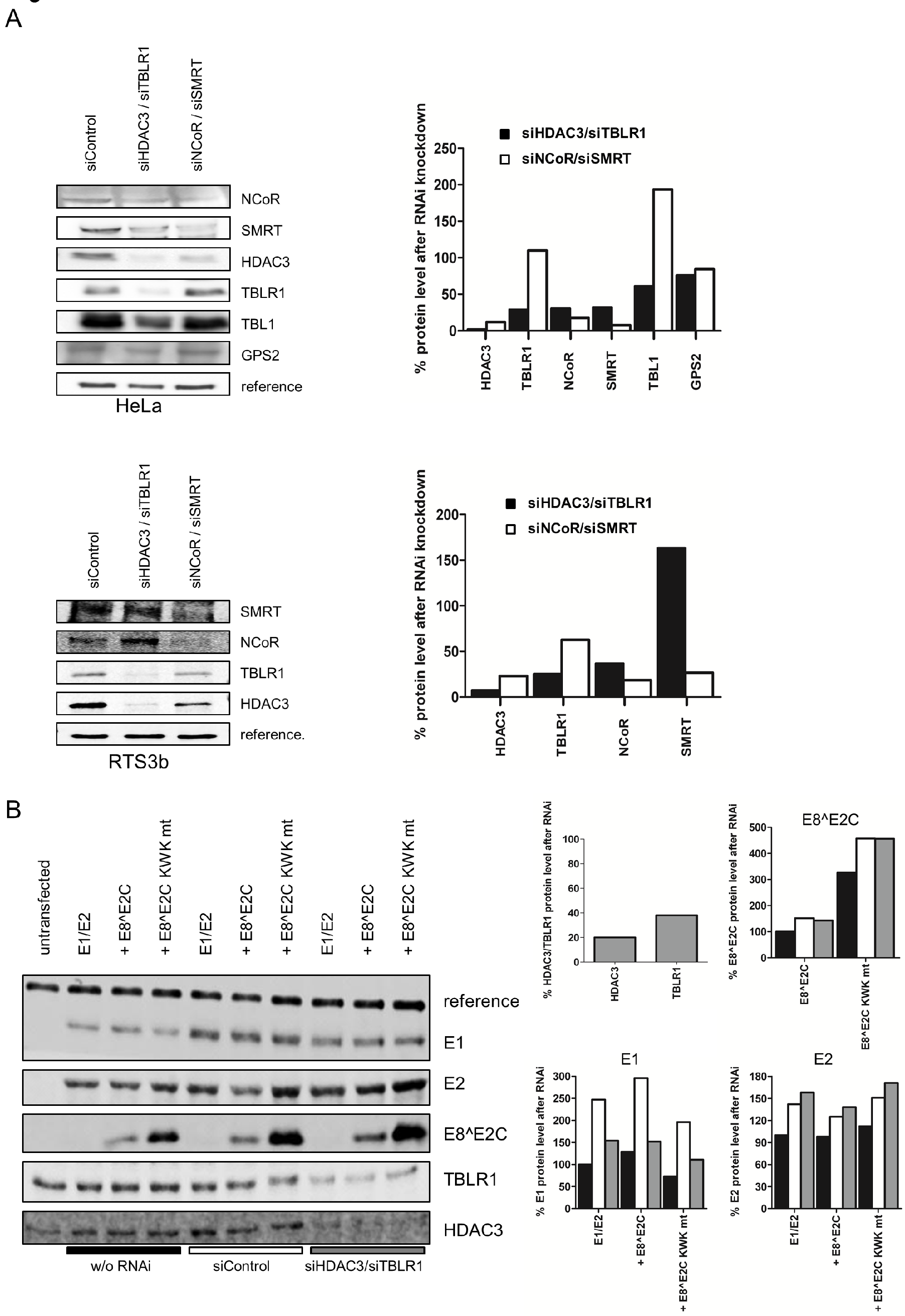

Supplement: S5 Fig — (A) HeLa or RTS3b were transfected with siRNA combinations (80pmol of each siRNA in HeLa cells and 166pmol of each siRNA in RTS3b cells) against HDAC3 and TBLR1 or against NCoR and SMRT. (B) HeLa cells were either not transfected (untransfected) or transfected with the siControl or the combination against HDAC3 and TBLR1. Twenty-four h later the cells were transfected with expression vectors for HPV 31 Flag-E1 (500 ng), myc-E2 (500 ng) and HA-E8^E2C or the KWK mt. Nuclear extracts were prepared and analyzed by immunoblotting with the indicated antibodies. KRIP1 was used as the nuclear reference to perform a quantification of Immunoblot-signals with the protein levels of the “siControl” sample (for A) or the “w/o RNAi” sample (for B) set to 100%. (TIF) [file ppat.1005556.s005.tif]
